# Early induction of the Rho-GEF ECT2 drives MEK/ERK oncogenic signaling in pancreatic ductal adenocarcinoma

**DOI:** 10.1038/s41388-026-03860-3

**Published:** 2026-06-19

**Authors:** Dania Al-Qasrawi, Nayya N. Murray, Ryan A. Argo, Alicia K. Fleming Martinez, Prita Pandya, Anaya Y. Clarke, Kayla C. Winter, Murli Krishna, Peter Storz, Nicole R. Murray, Verline Justilien

**Affiliations:** 1 Department of Cancer Biology, Mayo Clinic, Jacksonville, FL USA; 2 Comprehensive Cancer Center, Mayo Clinic, Jacksonville, FL USA; 3 Mayo Clinic Graduate School of Biomedical Sciences, Jacksonville, FL USA; 4 University of North Florida, Jacksonville, FL USA; 5 Department of Pathology/Lab Medicine, Mayo Clinic, Jacksonville, FL USA

**Keywords:** Pancreatic cancer, RHO signalling

## Abstract

Pancreatic ductal adenocarcinoma (PDAC) is among the deadliest cancers because it is typically detected at an advanced stage, progresses rapidly, and resists current therapies. Consequently, early diagnostic biomarkers and novel therapeutic targets are urgently needed. Epithelial Cell Transforming Sequence 2 (ECT2) is a Rho family guanine nucleotide exchange factor that was originally identified as an oncoprotein and later shown to regulate cytokinesis. Here, we evaluated ECT2 expression in human PDAC and its functional requirement for transformed growth and tumorigenicity. We found that ECT2 expression is elevated early in PDAC tumorigenesis, remains high throughout progression, and correlates with poor patient survival. Furthermore, a significant pool of ECT2 is mis-localized in the cytoplasm of PDAC cells. Knockdown of ECT2 inhibited 3D-transformed growth, invasion, and in vivo tumor formation while having little impact on PDAC cell cytokinesis. Mechanistically, we found that ECT2 is required for activation of Rac1 and RhoA and downstream MEK/ERK and ROCK signaling, respectively. Consistent with these findings, analyses of PDAC patient datasets revealed a strong association between ECT2 expression and Rho GTPase as well as MEK/ERK and ROCK pathway signatures. Finally, genetic or pharmacologic targeting of ECT2 signaling enhanced PDAC cell sensitivity to MEK inhibition. Taken together, our data identify ECT2 as an early driver of PDAC transformation and highlight it as a promising therapeutic target.

## Introduction

Pancreatic ductal adenocarcinoma (PDAC) accounts for greater than 90% of pancreatic cancer cases, the third leading cause of cancer-related deaths in the United States [1]. PDAC prognosis remains poor with a 5-year overall survival rate of 13% which falls to 3% for the majority of patients diagnosed with metastatic disease [1]. Due to a lack of reliable biomarkers and sensitive imaging modalities for early detection of PDAC, more than 80% of patients present with locally advanced or metastatic cancers that are often resistant to current therapies at the time of diagnosis [2]. A deeper understanding of the molecular mechanisms of PDAC maintenance and progression is critically important for the discovery of early diagnostic biomarkers and novel therapeutic targets.

*KRAS* is the most frequently mutated oncogene in PDAC, with activating mutations detected in over 90% of PDAC cases [3]. *KRAS* mutations which are most commonly found at codon 12, lead to constitutive activation of KRAS and chronic stimulation of RAF-MEK-ERK and PI3K-AKT-mTOR pathways, thereby promoting PDAC cell proliferation, survival, metabolic reprogramming, immune evasion, and therapeutic resistance [4–7]. KRAS also signals through activation of the Rho family GTPases (RhoA, Rac1, and Cdc42) [8]. These GTPases are activated by guanine nucleotide exchange factors (GEFs), which catalyze GDP-GTP exchange to initiate downstream effector signaling. Aberrant activation of Rho-GEFs such as VAV1, TIAM1 and DOCK8 drives cytoskeletal remodeling, cell migration, invasion and epithelial-to-mesenchymal transition, processes which promote PDAC progression [9–11].

Epithelial cell transforming sequence 2 (ECT2) is unique among the Rho-GEFs, exhibiting predominantly nuclear expression during interphase, and performing an essential, non-redundant role in cytokinesis [12]. We previously identified ECT2 as an oncogenic KRAS effector in lung adenocarcinoma (LUAD), where it plays a required role in *Kras-Trp53*-driven tumor initiation in vivo by sustaining a highly tumorigenic population of tumor-initiating cells and by activating proliferative signaling [13]. Additional studies have implicated ECT2 in ovarian, colorectal and liver cancers [14–16]. Although ECT2 expression has been found to be elevated in PDAC tumors [17, 18], whether ECT2 also mediates oncogenic KRAS signaling in PDAC has not yet been explored.

In this study, we show that ECT2 is overexpressed early in PDAC progression and is required for maintaining transformed growth, invasion, stemness, and tumorigenicity. Mechanistically, ECT2 regulates MEK/ERK and ROCK signaling through Rac1 and RhoA activation, respectively, linking it to oncogenic KRAS signaling. Notably, genetic or pharmacologic targeting of ECT2 signaling enhanced PDAC cell sensitivity to MEK inhibition. Pathway analysis further supports a clinical association between high ECT2 expression and Rho GTPase, ERK/MAPK and ROCK signaling highlighting ECT2 as a potential biomarker and therapeutic target in PDAC.

## Materials and methods

### Cell lines, antibody reagents, and inhibitors

List of antibodies used is in Table S1. Human pancreatic cancer cell lines obtained from the American Type Culture Collection (ATCC, Manassas, VA, USA) (AsPC1 (RRID:CVCL_0152), BxPC3 (RRID: CVCL_0186), CAPAN1 (RRID: CVCL_0237), CAPAN2 (RRID: CVCL_0026), CFPAC-1 (RRID: CVCL_1119), HPAF-II (RRID: CVCL_0313), Hs766T (RRID: CVCL_0313), MiaPaCa-2 (RRID:CVCL_0428), and PANC-1 (RRID:CVCL_0480)) obtained from ATCC along with human pancreatic ductal epithelial cells HPDE (RRID: CVCL_4376) were generously provided by Dr. Peter Storz (Mayo Clinic, FL). Experimental cells were sourced from the original stocks stored in liquid nitrogen. Cancer cell lines were cultured in RPMI 1640 (RRID: SCR_013297), ISCOVE (RRID: SCR_013311) or DMEM Medium (RRID: SCR_010885) (Invitrogen-Thermo Fisher Scientific, Waltham, MA, USA), supplemented with 10% fetal bovine serum, 100 U/ml penicillin, and 100 mg/ml streptomycin (Invitrogen-Thermo Fisher Scientific), maintained at 37 °C with 5% CO_2_ as recommended by ATCC. HPDE cells were cultured as described previously [16]. Cells were monitored for Mycoplasma contamination using the Lonza MycoAlert Mycoplasma Detection Kit (Lonza, Walkersville, MD).

Auranofin was obtained from Santa Cruz Biotechnology (Dallas, TX, USA). Trametinib, NSC23766 and Y-27632 were obtained from MedChemExpress (Monmouth Junction, NJ, USA). Inhibitors were dissolved in 100% dimethyl sulfoxide (DMSO; Sigma-Aldrich, St. Louis, MO, USA) and diluted with cell culture media to obtain a final DMSO concentration of 0.1% for experiments. DMSO was used at 0.1% (v/v) as vehicle control.

### Primary PDAC analysis

#### Biospecimens

RNA was isolated from a set of previously reported paired PDAC tumor and non-tumor pancreas tissues obtained from the Mayo Clinic Tissue Registry under an approved Institutional Review Board (IRB) protocol [19]. Formalin-fixed, paraffin-embedded non-tumor pancreas, PanIN, and PDAC tissues were sourced from archived materials at Mayo Clinic under an approved IRB protocol. Clinicopathological features are summarized in Table S3. Laser capture microdissection, described previously [20], was used to isolate PanIN and tumor lesions from formalin-fixed, paraffin embedded tissue. Tissues from *LSL-Kras*^*G12D*^;*LSL-p53*^*R172H*^;*Pdx1*^*cre/+*^ mice were previously described [20].

#### Publicly available datasets

RNA-seq and clinical data for primary PDAC tumors were obtained from The Cancer Genome Atlas (TCGA) via cBioPortal (http://www.cbioportal.org/public-portal/) and UCSC Xena (https://xenabrowser.net/). Normal pancreas RNA-seq data were retrieved from the Genomic Data Commons (GDC; https://portal.gdc.cancer.gov/). Publicly available RNA-seq datasets were also obtained from the Gene Expression Omnibus (GEO; https://www.ncbi.nlm.nih.gov/geo/), and protein expression data were obtained from the Clinical Proteomic Tumor Analysis Consortium (CPTAC; https://proteomics.cancer.gov/programs/cptac) via the UALCAN portal (https://ualcan.path.uab.edu/analysis-prot.html) .

#### GISTIC analysis

Copy number alterations in the TCGA PDAC (PAAD) dataset were assessed using the Genomic Identification of Significant Targets in Cancer (GISTIC) algorithm. GISTIC scores were then compared with ECT2 mRNA expression levels to examine correlations between gene expression and genomic copy number status.

#### Survival analysis

Overall survival of PDAC patients from the TCGA dataset was evaluated after stratifying tumors by ECT2 expression, with the highest and lowest 25% of cases used for comparison. Kaplan–Meier survival curves were generated in GraphPad Prism.

#### Ingenuity pathway analysis

Differential expression analysis was performed on TCGA PDAC tumors stratified by ECT2 expression, with the highest 30% and lowest 30% of cases compared. Genes with a fold change >1.5 and *q*-value < 0.05 were selected and used as input for Ingenuity Pathway Analysis (IPA, Qiagen Redwood City, CA). The core analysis was performed using default parameters, considering both direct and indirect experimentally validated interactions from the Ingenuity Knowledge Base. Canonical pathways were filtered using –log(p-value) >1.3, together with a z-score threshold of ≥2.

#### Gene set enrichment analysis (GSEA)

GSEA was performed on the TCGA PDAC dataset by stratifying tumors according to ECT2 expression, with the highest 30% and lowest 30% of cases compared. Enrichment analysis was conducted to identify gene sets significantly associated with ECT2 expression, using default parameters and curated gene sets from the Molecular Signatures Database (MSigDB).

#### Pathway signature scores

In the TCGA PDAC dataset, the ERK pathway score was calculated from genes significantly enriched in the GSEA results and evaluated for association with ECT2 expression as well as other GEFs. The KRAS–ERK score was determined using a previously reported gene signature [21] and analyzed against ECT2 expression. In addition, a ROCK pathway signature score was derived from established ROCK target genes and examined for correlation with ECT2 and other GEFs. The corresponding gene sets are summarized in Table S3.

#### Immunohistochemical analysis of human primary PDAC tumors

Formalin-fixed, paraffin-embedded blocks with adjacent non-tumor pancreas, pancreas tissue with PanIN lesions or primary PDAC tumor were cut into 5 μm sections, baked, deparaffinized for 15 min in xylene, rehydrated through graded alcohols, and washed with distilled water. Antigen retrieval was performed in citrate buffer (pH 6.0) for 25 min at 100 °C. Slides were washed in tap water followed by phosphate-buffered saline containing 0.5% Tween-20, then treated with 3% hydrogen peroxide for 5 min to block endogenous peroxidase activity. Sections were subsequently incubated for 60 min at room temperature with antibodies against ECT2 or Ki67. Slides were washed, incubated with secondary polymer detection reagent, developed with DAB, counterstained in hematoxylin, dehydrated, and mounted. Slides were scanned at ×20 magnification using an Aperio ScanScope system (Leica Biosystems, Buffalo Grove, IL). ECT2 and Ki67 staining analysis were performed using the Aperio ScanScope software. Briefly, regions of interest (ROI) containing normal, PanIN or PDAC tumor tissues were selected in each case, scored for staining intensity (0, 1+, 2+, and 3+) and extent of staining (% tumor cells staining at each intensity) was used to generate a histoscore ranging from 0 to 300 as described previously [22].

### Lentiviral RNAi, expression constructs, cell transduction, qPCR, and immunoblot analysis

Lentiviral vectors containing human ECT2 shRNAs or a non-targeting control shRNA (Table S1) were acquired from Sigma (MilliporeSigma, Rockville, MD), packaged into recombinant lentivirus, and utilized to establish stable cells as described previously [17]. cDNAs for human HA-tagged RNAi-resistant wildtype, GEF-deficient and NLS mutant ECT2 were described previously [13]. ECT2 cDNAs were packaged into lentiviral vectors according to established methods [16], and cells stably transduced with lentiviral ECT2 cDNAs were generated as described previously [10]. Myc-caRac1V12 (caRac1) and Myc-caRhoA-V14 (caRhoA) expression constructs were kind gifts from Dr. P. Z. Anastasiadis (Mayo Clinic, FL) [23]. Total RNA was isolated from cells using the RNAeasy Plus Mini Kit (QIAGEN) followed by cDNA conversion (Bio-Rad, Hercules, CA, USA). The efficiency of ECT2 knockdown and 45S pre-RNA abundance was assessed by qPCR using TaqMan or SYBR green, respectively as described previously [10]. Transcript levels were normalized to GADPH or B-Actin, respectively. Primer sets are listed in Table S1. Immunoblot analysis was performed as previously described [13]. Briefly, cells were lysed in RIPA buffer (10 mM Tris pH 7.5, 150 mM NaCl, 1 mM EDTA, 0.5% sodium deoxycholate, 0.1% SDS, 1% Triton X-100) supplemented with protease and phosphatase inhibitors (Thermo Fisher Scientific, Waltham, MA, USA). Lysates were sonicated and protein concentrations determined using a BCA Protein Assay Kit (Thermo Fisher Scientific, Waltham, MA, USA). Lysates were mixed with 4X Laemmli sample buffer and reducing agent, boiled, and equal amounts of protein (10–20 μg) were resolved on 4–20% SDS-polyacrylamide gels (Invitrogen, Thermo Fisher Scientific, Waltham, MA, USA). Proteins were transferred to polyvinylidene difluoride membranes (Immobilon-P, Millipore, Burlington, MA, USA). Membranes were blocked in 5% nonfat dry milk in TBS-Tween 20, incubated with the indicated primary antibodies, and probed with HRP-conjugated secondary antibodies (SeraCare Life Sciences, Milford, MA, USA). Signals were detected using ECL-Plus (Amersham Biosciences, GE Healthcare, Chicago, IL, USA). Densitometry analysis for protein bands was performed using the ImageJ software (ImageJ, RRID:SCR_003070).

### Soft agar colony formation and invasion assays

PDAC cells were plated in soft agar and assessed for anchorage-independent/transformed growth as described previously [24]. Complete 2X growth medium was mixed with 1.5% agarose at a 1:1 ratio to generate a bottom layer of soft agar (final concentration 0.75%) in 35mm-well tissue culture dishes. Single-cell suspensions containing 3000 cells per well were prepared in 0.5% agarose (SeaKem GTG Agarose, Lonza Basel, Switzerland) mixed with 2X medium and layered over the solidified bottom agar. For Auranofin, Trametinib, NSC23766 and Y-27632 dose response experiments, drugs were added to both the bottom and top agar layers at the concentrations indicated in the figures. Plates were incubated at 37 °C in 5% CO₂, and colony formation was assessed after 3–4 weeks, depending on the cell line. Colonies were fixed in methanol for 20 min at room temperature, washed twice with 1X PBS, and stained with Giemsa (1:20 dilution in PBS; EMD Millipore, Burlington, MA, USA) for 2 h. Stained plates were washed with PBS, imaged under light microscopy, and colony size and number were quantified using ImageJ (ImageJ, RRID:SCR_003070).

Cellular invasion was measured in transwell chambers coated with Matrigel basement membrane (Corning Costar, Corning, NY, USA) as described previously [24]. Inserts were hydrated in serum-free medium for 2 h at 37 °C prior to cell seeding. A total of 2 × 10⁵ PDAC cells suspended in serum-free medium were added to the upper chamber, and 750 μL of medium supplemented with 5% FBS was placed in the lower chamber to serve as chemoattractant. After 20 h of incubation at 37 °C in 5% CO₂, non-invading cells on the upper surface of the membrane were removed with a cotton swab. Invaded cells on the lower membrane surface were fixed in methanol at −20 °C for 30 min and stained with MES–crystal violet. Images of invaded cells were captured using light microscopy and quantified using ImageJ software (ImageJ, RRID:SCR_003070).

### Oncosphere formation and clonal expansion assay

PDAC cancer stem cells (CSCs) were enriched in DMEM-F12 medium (Gibco-Invitrogen, Thermo Fisher Scientific, Waltham, MA, USA) supplemented with 50 mg/ml insulin (MilliporeSigma), 0.4% Albumin Bovine Fraction V (MilliporeSigma), N-2 Plus Media Supplement (Thermo Fisher Scientific), B-27 Supplement (Gibco-Invitrogen- Thermo Fisher Scientific), 20 ng/ml EGF (PeproTech, Rocky Hill, NJ, USA), and 10 ng/ml bFGF (PeproTech) in ultra-low attachment flasks (Corning) to facilitate the growth of undifferentiated oncospheres [12]. For clonal expansion, single cells were seeded into each well of 96-well ultra-low attachment tissue culture plates (Corning), and clonal expansion was assessed after 14–21 days as described previously [24].

### MTT assays and population doubling time analysis

2000 PDAC cells per well were plated in 96-well plates. Cell viability was determined at one, three, five and seven days after plating using the CellTiter-Glo luminescent cell viability kit from Promega Corporation (Madison, WI, USA) according to the manufacturer’s instructions. For Auranofin, Trametinib, NSC23766 and Y-27632 dose response, 2,500 cells per well were seeded in 96-well plates and treated the following day with concentrations of inhibitors as indicated in the figures. Cell viability was measured after 72 hours of treatment. Population doubling time (PDT) was determined during logarithmic growth phase using the formula PDT=ln2t/ln(N/No) (N=final cell number, No=initial cell number and t=time between No and N).

### Immunofluorescence staining, microscopy and multinucleated cell analysis

PDAC cells were seeded onto sterile glass coverslips (Corning Inc., Corning, NY, USA) and cultured under standard growth conditions until ~70% confluency. Cells were fixed in 4% paraformaldehyde (Electron Microscopy Sciences, Hatfield, PA, USA) for 15 min at room temperature, permeabilized with 0.1% Triton X-100 (Sigma-Aldrich, St. Louis, MO, USA) for 10 min and blocked in 5% bovine serum albumin (BSA; Sigma-Aldrich, St. Louis, MO, USA) in PBS for 1 h. For cytoskeletal staining, cells were incubated with Phalloidin–Alexa Fluor 594 (Thermo Fisher Scientific, Waltham, MA, USA) for 30 min, rinsed, and mounted using Vectashield mounting medium with DAPI (Vector Laboratories, Burlingame, CA, USA) to visualize nuclei, as described previously [25]. Representative fields were photographed at ×20 magnification, and more than 700 cells per cell type were analyzed for multinucleation.

For ECT2 localization studies, PDAC cells on coverslips were fixed, permeabilized, and blocked as above, then incubated overnight at 4 °C with an anti-ECT2 rabbit primary antibody (1:200 dilution; Millipore, Burlington, MA, USA) diluted in 1% BSA/PBS. After three washes with PBS, cells were incubated for 1 h at room temperature with an Alexa Fluor 488–conjugated anti-rabbit IgG secondary antibody (1:500 dilution; Thermo Fisher Scientific, Waltham, MA, USA). Following extensive washing, coverslips were mounted in Vectashield mounting medium with DAPI. Confocal images were acquired using a Zeiss LSM 510 META confocal microscope with Zeiss imaging software and a EC Plan-Neofluar 40X oil immersion objective lens (1.3 numerical aperture; Carl Zeiss Inc., Maple Grove, MN, USA), as described previously [13].

### Subcellular fractionation

Cells were fractionated using NE-PER Nuclear and Cytoplasmic Extraction Reagents (Thermo Fisher Scientific) as described previously [25]. Briefly a total of 2 × 10⁶ cells were used per fractionation. Cells were sequentially treated with Cytoplasmic Extraction Reagent I (CER I) and Cytoplasmic Extraction Reagent II (CER II) to release cytoplasmic proteins, followed by additional buffer extraction and centrifugation to isolate the nuclear fraction. Nuclear and cytoplasmic fractions were subjected to immunoblot analysis as described above using antibodies to ECT2, HA, Lamin A/C, and MEK1. Relative abundance of ECT2 in each fraction was determined by densitometry.

### Rho GTPase activity assays

Rho GTPase activity was determined using GST-tagged PAK-PBD (MilliporeSigma) (for Rac1 and Cdc42) or Rhotekin (MilliporeSigma) (for RhoA) pull-down assays for active (GTP-bound) GTPase in cell lysates as described previously [25]. Briefly, cells were lysed in a lysis buffer (e.g., 50 mM Tris-HCl, pH 7.4, 150 mM NaCl, 1% NP-40, protease inhibitors) and protein concentrations were quantified using BCA. Equal amounts of protein from each sample were incubated with GST-tagged PAK-PBD or Rhotekin, which specifically bind to the active, GTP-bound form of the GTPase, and the mixtures were incubated overnight at 4°C. The complexes were then washed three times with cold lysis buffer to remove any non-specific binding. The bound active GTPases were eluted by 2x Dye. Active Rho GTPase pull down were assessed by immunoblotting and densitometry. Results were normalized to total Rho GTPase.

### Tumorigenicity in NOD-SCID mice

All animal experiments were performed under the approved Mayo Clinic Institutional Animal Care and Use Committee IACUC protocol (A00006924-22). 1 × 10^6^ PANC-1 or MiaPaCa-2 shNT and shECT2 cells were suspended in 50% Growth Factor Reduced Matrigel Matrix (BD Biosciences, San Jose, CA, USA) in 1X-PBS and subcutaneously injected into the right flank of male and female 6–8 week old NOD-SCID mice purchased from Jackson laboratory, (Bar Harbor, ME) using a 28-gauge needle in a final volume of 50 µL as described previously [26]. The mice were randomly divided into the three experimental tumor cell inoculation groups as described in figure legend. Tumor growth was monitored by caliper measurement and tumor volume was estimated using the formula 0.5236 (*L* × *W* × *H*), where *L* represents the length of the tumor, *W* represents the width of the tumor, and *H* represents the height as described previously [25]. After 6 weeks, mice were harvested, tumors collected, and final tumor weights were determined. The number of mice used were to provide adequate statistical power to determine a two-fold change in a tumor parameter across groups, and to provide sufficient tissue for molecular analyses. We estimate the number of mice to be used based on our own published literature [13, 19] and ANOVA statistical analyses for sample size (desired power = 0.8; alpha = 0.05).

A separate cohort of NOD-SCID mice were injected with 50 uL of 1 × 10^6^ PANC-1-luciferase shNT or shECT2 cells in the proximal pancreas as previously described [19]. For in vivo imaging, mice were anesthetized with isoflurane and injected intraperitoneally with D-luciferin (15 mg/kg) and observed using an IVIS system (Caliper Life Sciences, Waltham, MA). After 5 weeks, mice were euthanized, and tumors were harvested. Abdominal and chest organs were evaluated for metastatic tumor formation.

PDAC tumors derived from both flank injections and orthotopic implantation of PDAC cells were fixed in 10% neutral-buffered formalin and embedded in paraffin. Sectioned slides were processed and stained for ECT2, Ki67, CD31, ERK, and p-ERK as described above for primary PDAC tumors. A positive pixel algorithm was applied to each ROI in the Aperio Slide Imaging software, and the resulting number of positive pixels was divided by the total number of pixels to generate the percent positivity. Lysates from harvested tumors were prepared by homogenization in RIPA lysis buffer supplemented with protease and phosphatase inhibitor cocktails (Sigma-Aldrich) as described previously [25] and immunoblot analyses for ERK1,2, phospho-ERK1,2 and B-Actin were carried out as described above. No animals or samples were excluded unless they failed to meet predefined quality-control criteria (e.g., tumor engraftment failure). Both tumor cell inoculations and growth measurements were performed by the same investigators.

### Drug interaction studies

The effect of drug combinations was evaluated by exposing the cells to different concentrations of drugs and analyzing results using the median effect combination index (CI) of Chou and Talalay, calculated using CompuSyn software (ComboSyn Inc.). CI values lower than 0.90 indicate synergistic drug interactions; conversely, CI values higher than 1.20 indicate antagonism, and values between 0.90 and 1.20 were considered additive.

### Statistical analysis

Statistical significance of results was analyzed using the GraphPad Prism (GraphPad Prism, RRID:SCR_002798) program version 9. Statistical differences were analyzed using the Student paired two-tailed t-test, one-way ANOVA, and chi-squared analysis. *P*-values less than 0.05 were considered significant.

## Results

### ECT2 overexpression occurs early in PDAC tumorigenesis

**Fig. 1:**
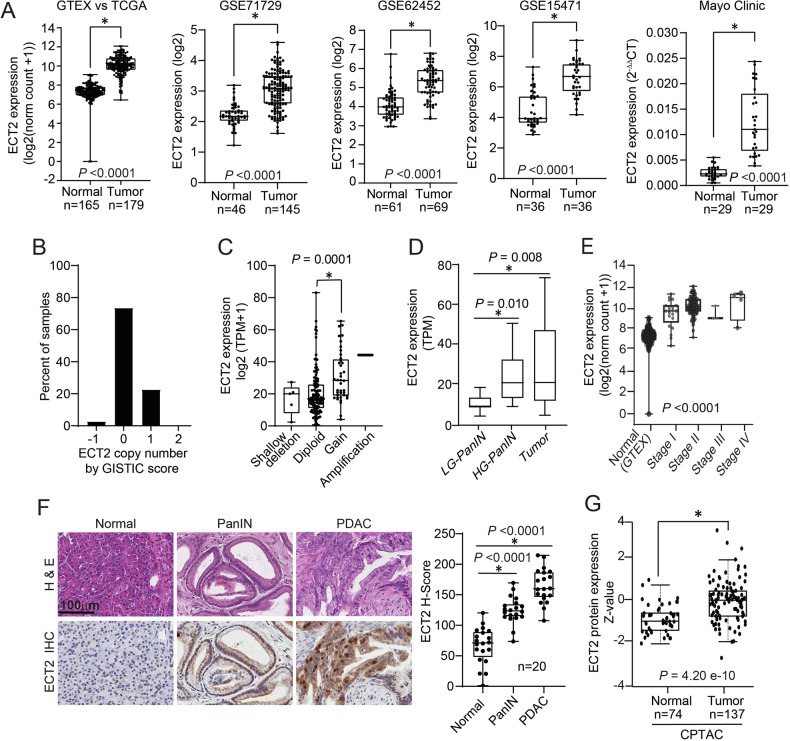
ECT2 is highly expressed in PDAC. **A** Comparison of ECT2 mRNA levels between PDAC and non-tumor pancreas tissues from The Cancer Genome Atlas (TCGA) and GTEx datasets (left panel), GEO datasets (center panels) and from the Mayo Clinic Tissue Registry (right panel). **B** GISTIC analysis for *ECT2* gene copy number alteration (shallow deletion=GISTIC score, −1; diploid=GISTIC score, 0; gain = GISTIC score, +1; amplification = GISTIC score, 2) (−1, *n* = 5; 0, *n* = 129; +1, *n* = 40; +2, *n* = 1). **C** ECT2 mRNA expression in TCGA PDAC tumors by GISTIC score of the *ECT2* gene. **D** ECT2 mRNA expression in laser microdissected low grade PanIN, high grade PanIN and PDAC lesions. *n* = 10. **E** ECT2 mRNA expression in PDAC tumors by clinical stages (GTEx Normal, *n* = 167; stage I, *n* = 21; stage II, *n* = 146; stage III, *n* = 3; stage IV, *n* = 4). **F** Representative hematoxylin and eosin (H&E) and ECT2 IHC images (left panel) and plot of ECT2 histoscore (right panel) in human non-tumor pancreas, PanIN, and PDAC. **G** ECT2 protein levels in PDAC tumors and non-tumor pancreas tissue in the CPTAC database.

To investigate the role of ECT2 in PDAC, we compared PDAC tumor samples from The Cancer Genome Atlas (TCGA) PAAD dataset with normal pancreas from the Genotype-Tissue Expression (GTEx) project, which revealed that ECT2 mRNA expression is significantly elevated in PDAC tissues (*left panel*; Fig. 1A). Similarly, elevated levels of ECT2 were observed in three publicly available RNA-seq datasets from the Gene Expression Omnibus database (center panels; Fig. 1A). qPCR analysis of 29 paired primary PDAC tumors and adjacent non-tumor pancreas tissue from the Mayo Clinic Tissue Registry [19] confirmed increased ECT2 mRNA in tumors (*right panel*; Fig. 1A). *ECT2* is often amplified as part of the chromosome 3q26 region in tumors, therefore, we also examined *ECT2* copy number status in PDAC samples. GISTIC analysis of the TCGA PDAC cohort revealed rare high copy number gain/amplification (CNG; score +2), low-level gain (score +1) in ~20% and shallow loss (score −1) in ~3% of tumors (Fig. 1B). PDAC tumors with *ECT2* CNG expressed significantly more ECT2 mRNA than diploid tumors, but most tumors lacking CNG also displayed high ECT2 (Fig. 1A), indicating additional regulatory mechanisms of ECT2 overexpression.

PDAC develops through stepwise morphological progression in which normal exocrine pancreatic epithelial cells undergo metaplasia followed by transition to increasing grades of preneoplastic lesions such as pancreatic intraepithelial neoplasias (PanINs), and finally invasive PDAC [17]. Interrogation of ECT2 mRNA expression from microdissected low-grade PanIN, high-grade PanIN, and PDAC samples from a panel of 10 patients, revealed that ECT2 becomes significantly elevated from low- to high-grade PanIN stages and remained high at the PDAC stage (Fig. 1D). Furthermore, ECT2 overexpression was equally prevalent in stage I and later stage tumors (Fig. 1E) suggesting that ECT2 may be important for the maintenance of PDAC tumors.

Next, we determined whether increased ECT2 mRNA abundance results in elevated ECT2 protein expression in PDAC tumors. Immunohistochemistry (IHC) of 20 paired adjacent normal pancreas, PanIN, and PDAC lesions showed stronger ECT2 staining in tumor cells than in non-tumor epithelium (Figs. 1F and S1A). Normal epithelial staining for ECT2 was nuclear, whereas ECT2 was detected in both the nucleus and cytoplasm of tumor tissue (Fig. 1F). Consistent with our observation at the mRNA level, ECT2 IHC staining was also elevated early in PanIN lesions (Figs. 1F and S1A). Clinicopathologic characteristics of patients are summarized in Table S3. Higher ECT2 protein expression in PDAC was validated through analysis of 137 PDAC and 74 normal pancreas samples in the CPTAC database (Fig. 1G). Likewise, Ect2 staining was induced in PanIN lesions and PDAC tumors that develop in *LSL-Kras*^*G12D/+*^*;LSL-Trp53*^*R172H/+*^*;Pdx-Cre* mice relative to normal mouse pancreas (Fig. S1B), indicating that ECT2 protein is generally elevated in PDAC. Taken together, our results demonstrate that ECT2 overexpression is an early event in PDAC development.

### ECT2 is required for PDAC-transformed phenotypes

**Fig. 2:**
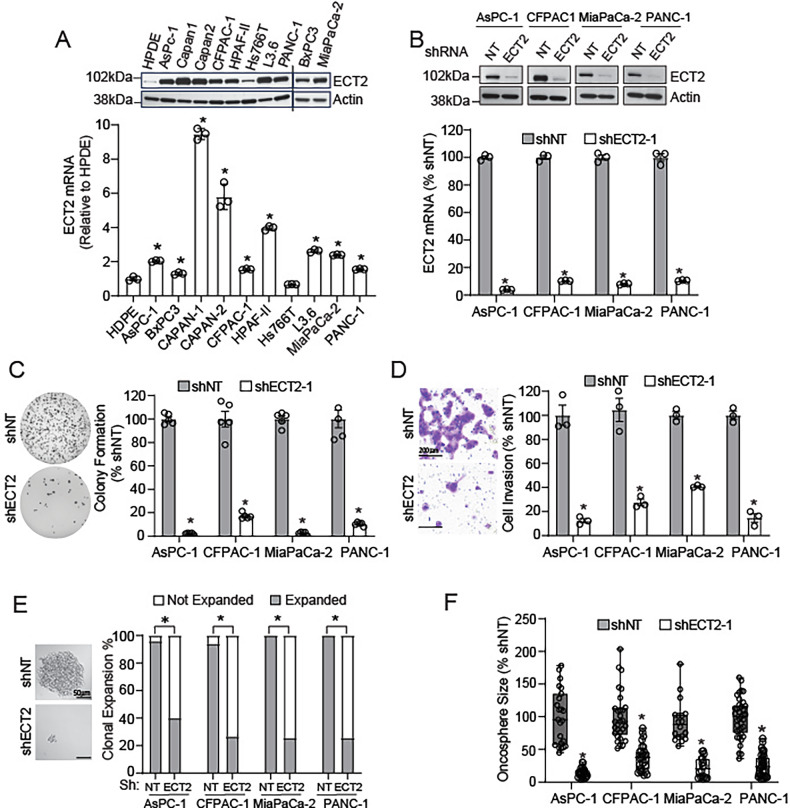
ECT2 is required for PDAC transformed phenotypes. **A** qPCR and immunoblot analysis of ECT2 mRNA and protein abundance in a panel of PDAC cell lines; *n* = 3. **B** qPCR and immunoblot analysis of ECT2 mRNA and protein abundance in AsPC-1, CF-PAC, MiaPaCa-2, and PANC-1 shECT2 knockdown cells; *n* = 3. **C** Representative photomicrographs and quantitation of soft agar colonies formed by PDAC shNT and shECT2 cells; *n* = 5. **D** Representative photomicrographs and quantitation of PDAC shNT and shECT2 cellular invasion through Matrigel-coated chambers; *n* = 3. **E** Representative photomicrographs of single PDAC shNT and shECT2 spheres and clonal expansion efficiency of PDAC CSCs in nonadherent culture. Results presented as % of single CSCs that expanded or did not expand; AsPC-1 (*n* = 25), CFPAC-1 (*n* = 30), MiaPaCa-2 (*n* = 30), and PANC-1 (*n* = 40). **F** Oncosphere size of PDAC shNT and shECT2 cells, normalized to the respective shNT control. Quantitated results in A-D and F expressed as % NT control and represent the mean ± SEM. (*) *p* < 0.05 compared to NT.

Human PDAC cell lines express elevated ECT2 relative to non-transformed human pancreatic ductal epithelial (HPDE) cells. (Fig. 2A). The prevalent overexpression of ECT2 in primary PDAC tumors and cell lines prompted us to directly assess the importance of ECT2 for PDAC maintenance. We used two independent shRNAs to knockdown ECT2 in AsPC-1, CFPAC-1, MiaPaCa-2, and PANC-1 PDAC cells which was confirmed by qPCR and immunoblot analysis (Figs. 2B and S2A). ECT2 depletion markedly reduced anchorage-independent growth in soft agar (Figs. 2C and S2B) and inhibited invasion in Transwell Matrigel assays (Figs. 2D and S2C). Cancer stem-like cells (CSCs) enriched from PDAC lines formed smaller spheres and showed diminished clonal expansion after ECT2 knockdown (Figs. 2E, F and S2D, E). These results show that ECT2 supports transformed growth, invasion, and stemness in PDAC cells.

### ECT2 is required for PDAC cell tumorigenicity in vivo and correlates with proliferation

**Fig. 3:**
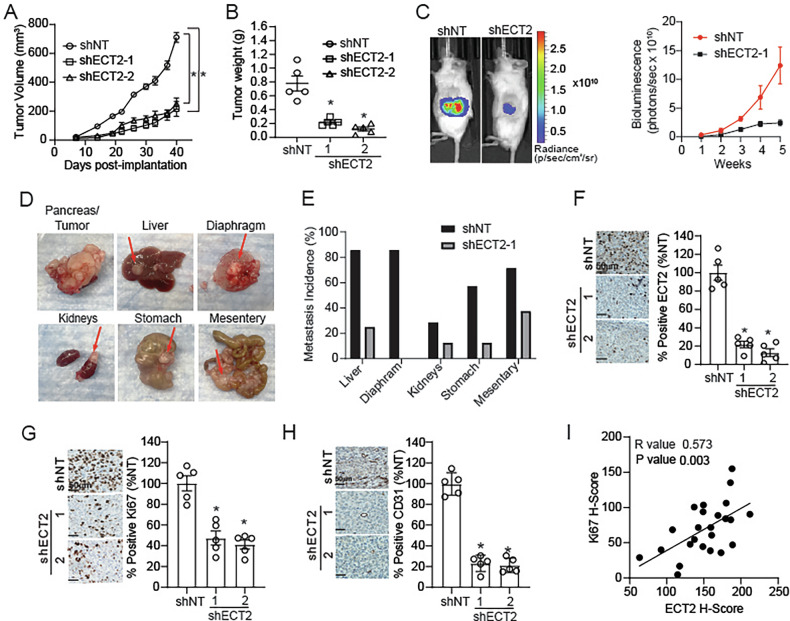
ECT2 is required for PDAC cell tumorigenicity in vivo and associates with PDAC cell proliferation in tumors. **A** Tumor growth curve of PANC-1 subcutaneous tumor bearing NOD-SCID mice inoculated with PANC-1 shNT or shECT2 cells, using two different ECT2 shRNA constructs (shECT2-1, and -2); *n* = 5 mice/group. **B** Final weights of tumors in panel A at harvest (42 days). **C** Representative bioluminescent images and growth of luciferase-expressing PANC-1 shNT and shECT2-1 cells orthotopically implanted into the pancreas of NOD-SCID mice; *n* = 7 mice/group. **D** Representative images and **E** percentage of metastasis incidence in different organs of mice bearing PANC-1 shNT or shECT2 orthotopic tumors. **F** ECT2, **G** Ki67 and **H** CD31 IHC in PANC-1 shNT or shECT2 subcutaneous tumors. Quantitated results represent the mean ± SEM and expressed as % NT control in **F**–**H**. **I** Scatter plot showing the association between ECT2 and Ki67 H-Scores in primary PDAC tissues; *n* = 23. (*), *p* < 0.05.

Next, to evaluate the requirement of ECT2 in PDAC tumorigenicity, we injected shNT or shECT2 PANC-1 and MiaPaCa-2 cells subcutaneously into the flanks of NOD-SCID mice and monitored tumor growth over the course of 6 weeks. shECT2 tumors grew at a slower rate than shNT tumors (Fig. 3A) and were smaller at 6-week harvest (Fig. 3B). Since ECT2 is required for PDAC cell invasion (Figs. 2C and S2B), a key step in metastasis, we examined whether ECT2 is required for metastasis. Orthotopic implantation of luciferase-expressing PANC-1 cells into the pancreas of NOD-SCID mice showed diminished tumor growth after ECT2 knockdown as measured by bioluminescent imaging (Figs. 3C and S3C). We observed frequent metastases in the liver, diaphragm, kidneys, stomach, and mesentery of mice with PANC-1 shNT orthotopic tumors which were decreased in shECT2 tumor-bearing mice (Figs. 3D, E and S3D). IHC confirmed sustained ECT2 depletion in shECT2 tumors (Figs. 3F and S3E, F) and revealed reduced Ki67 and CD31 staining in shECT2 tumors, indicating lower proliferation and vascularization (Figs. 3G, H and S3G–J). In resected human PDAC tumors, ECT2 histoscores correlated positively with Ki67 further supporting a role for ECT2 in highly proliferative tumors (Fig. 3I)*.* These data demonstrate that ECT2 is essential for PDAC tumor growth and metastasis in vivo.

### ECT2 knockdown does not impair anchorage-dependent growth of PDAC cells

**Fig. 4:**
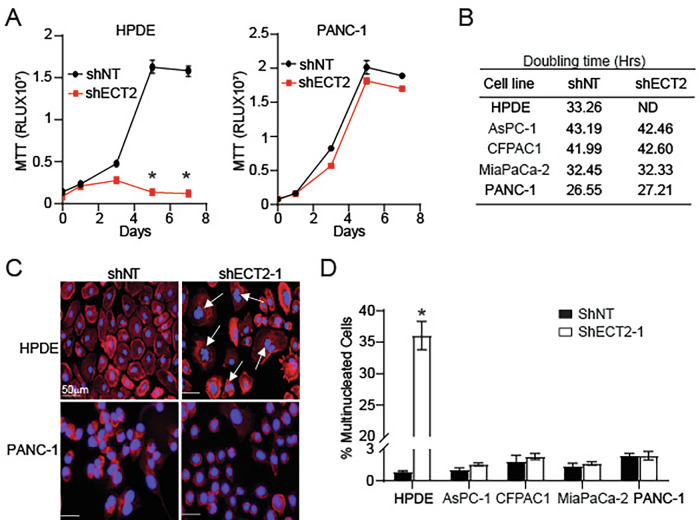
ECT2 knockdown does not impair PDAC anchorage-dependent cell growth. **A** Growth curve of normal HPDE pancreas cells and PANC-1 shNT and shECT2 cells over 7 days; *n* = 5 **B** Calculated population doubling time in hours for HPDE and PDAC shNT and shECT2 cells. **C** Representative DAPI (blue) and phalloidin (red) immunofluorescence to visualize multinucleated cells in HPDE and PDAC shNT and shECT2 cells. Multinucleated cells indicated by white arrows**. D** Quantitative analysis of multinucleated cells. n > 700. Results represent the mean ± SEM. (*) *p* < 0.05.

ECT2 is crucial for cytokinesis in non-transformed cells [27]. To determine whether ECT2 depletion impairs general PDAC cell division through defects in cytokinesis, we assessed cell proliferation, population doubling time, and multinucleated cells under 2D anchorage-dependent growth conditions. MTT assays showed that ECT2 knockdown sharply reduced proliferation of normal HPDE cells (Fig. 4A). Consistent with a decrease in cell proliferation, HPDE shECT2 cells exhibited an increase in population doubling time compared to HPDE shNT cells (Fig. 4B). HPDE shECT2 cells also accumulated multinucleated cells, indicative of a cytokinesis defect (Fig. 4C, D). In contrast, proliferation and doubling time of PDAC lines under 2D anchorage-dependent conditions were unaffected by ECT2 knockdown (Figs. 4A, B and S4A) and PDAC shECT2 cells did not accumulate multinucleated cells (Fig. 4C, D). These results indicate that the tumor-promoting functions of ECT2 in PDAC cells are uncoupled from its canonical cytokinetic role.

### ECT2 is abundant in both the nucleus and cytoplasm of PDAC cells

**Fig. 5:**
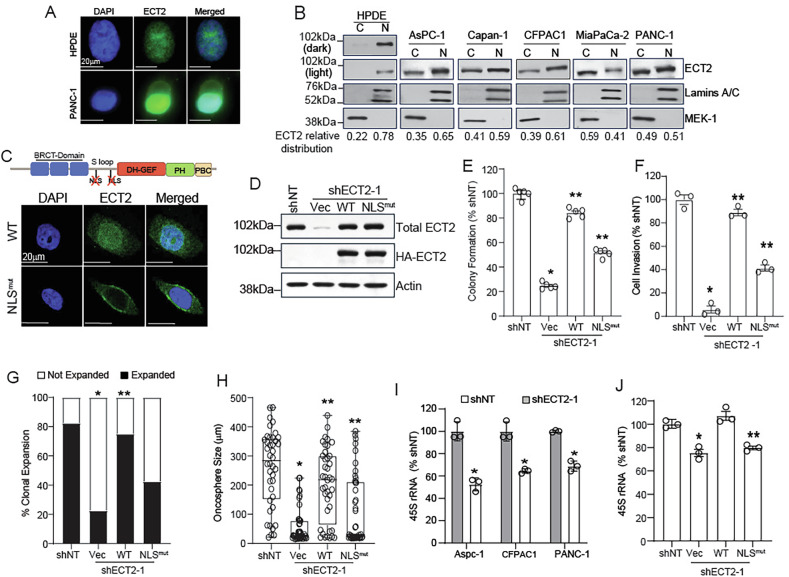
Nuclear and cytoplasmic ECT2 support PDAC transformation. **A** Representative immunofluorescence imaging of ECT2 (green) cellular localization in HPDE and PANC-1 cells. Nucleus is stained with DAPI. **B** Immunoblot analysis of ECT2 in cytoplasmic (C) and nuclear (N) fractions of PDAC and HPDE cell lines. Lamin A/C and MEK-1 served as control for the nuclear and cytoplasmic fractions, respectively. **C** Schematic of nuclear localization sequence-deficient ECT2 mutant (NLS^mut^) and immunofluorescent localization of HA-tagged wild-type (WT), and NLS^mut^ ECT2 in transfected PANC-1 cells. **D** Immunoblot analysis of PANC-1 shECT2 knockdown cells expressing HA-tagged WT, or NLS^mut^ ECT2 or vector (Vec). B-actin served as loading control. Effects of WT or NLS^mut^ ECT2 reconstitution in PANC-1 shECT2 knockdown cells on **E** transformed growth; *n* = 5 and **F** invasion through Matrigel-coated chambers; *n* = 3. Results expressed as % shNT and represent the mean ± SEM. **G** Effects of WT or NLS^mut^ ECT2 reconstitution in PANC-1 shECT2 knockdown cells on CSC clonal efficiency. Results presented as % of single CSCs that expanded or did not expand; *n* = 40. **H** CSC sphere size expressed as mean diameter in micrometers ± SEM; *n* = 40. **I** qPCR analysis of 45S rRNA in PDAC shECT2 knockdown cells. Results expressed as % shNT and represent the mean ± SEM. **J** qPCR analysis of 45S rRNA in PANC-1 shECT2 knockdown cells expressing vector (Vec), or WT, or NLS^mut^ ECT2. Results expressed as % NT and represent the mean ± SEM. (*) *p* < 0.05 compared to shNT and (**) *p* < 0.05 compared to vector in (**E**–**J**).

In non-transformed cells, ECT2 is expressed predominantly in the nucleus during interphase [27]. In various cancer cell types, a distinct pool of ECT2 has been shown to be mis-localized to the cytoplasm [16, 25, 28]. Immunofluorescence showed ECT2 in both cellular compartments in PDAC cells but predominantly nuclear in HPDE cells (Figs. 5A and S5A), consistent with IHC staining in primary human and mouse PDAC tumor cells (Figs. 1F and S2B). We also performed cell fractionation analysis to further assess ECT2 subcellular localization in PDAC cells. Fractionation and immunoblotting confirmed abundant nuclear and cytoplasmic ECT2 in PDAC lines whereas HPDE cells exhibited primarily nuclear localization (Fig. 5B).

### ECT2 cytoplasmic localization partially supports PDAC transformed phenotypes

Both nuclear- and cytoplasmic-localized ECT2 have been shown to play a role in cell transformation [13, 25]. To dissect compartment specific- roles, ECT2-depleted PANC-1 cells were reconstituted with an shRNA-resistant, hemagglutinin (HA)-tagged wildtype (WT) ECT2 or an HA-ECT2 mutant with alanine mutations of five key arginine residues (R348,349,350,370,372 A) in the ECT2 nuclear localization signal (NLS) (NLSmut-ECT2) (Fig. 5C). As expected, immunofluorescence and cell fractionation showed that HA-WT-ECT2 expressed in PANC-1 cells is localized to both the cytoplasm and nucleus whereas HA-NLSmut-ECT2 is predominantly cytoplasmic (Figs. 5C and S5B). We found that re-expression of HA-WT-ECT2 in shECT2 cells (Figs. 5D and S5C) could largely reconstitute PDAC cell transformed growth, invasion and spheroid growth, whereas HA-NLSmut-ECT2 provided only partial rescue (Figs. 5E–H and S5D–G). Since NLSmut-ECT2 could only partially reconstitute the transformed phenotype of PDAC shECT2 cells, we reasoned that nuclear localization of ECT2 also plays an important role in PDAC transformation. Nuclear ECT2 has been shown to regulate ribosomal RNA (rRNA) synthesis in lung and colon cancer cells which is required for the transformed growth of these tumor types [16, 29]. Therefore, we next assessed the effects of an ECT2 knockdownon 45S pre-ribosomal RNA (45S rRNA), a direct measure of rRNA synthesis. shECT2 PDAC cells showed a decrease in 45S rRNA abundance when compared with shNT cells (Fig. 5I) which could be reconstituted with WT-ECT2, but not the NLSmut-ECT2 (Fig. 5J). Thus, both nuclear and cytoplasmic ECT2 contributed to PDAC transformation, with nuclear ECT2 supporting ribosome biogenesis.

### ECT2 GEF activity is required for PDAC transformation

**Fig. 6:**
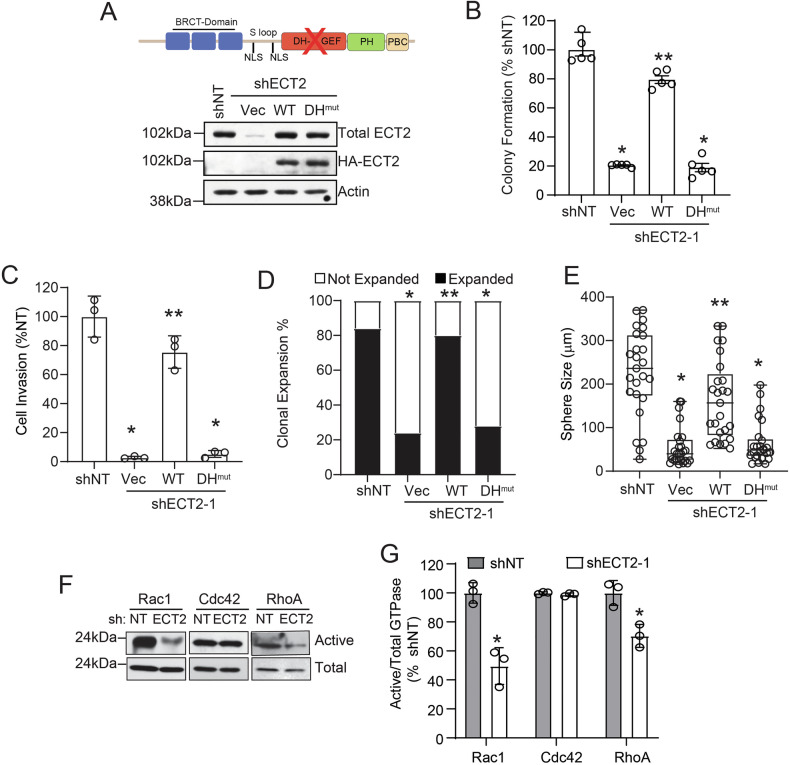
ECT2 GEF activity is required for PDAC transformation. **A** Schematic of GEF deficient (DH^mut^) and immunoblot analysis of PANC-1 shECT2 knockdown cells expressing HA-tagged vector (Vec) or WT, or DH^mut^ ECT2. B-actin served as loading control. Effects of WT or DH^mut^ ECT2 re-expression in PANC-1 shECT2 knockdown cells on (**B**) transformed growth; *n* = 5 and (**C**) invasion through Matrigel-coated chambers; *n* = 3. Results expressed as % shNT and represent the mean ± SEM. **D** Effects of WT or DH^mut^ ECT2 reconstitution in PANC-1 shECT2 knockdown cells on CSC clonal efficiency. Results presented as % of single CSCs that expanded or did not expand; n = 25. **E** CSC sphere size expressed as mean diameter in micrometers ± SEM; *n* = 25. **F** Representative immunoblots and **G** quantitative analysis of active and total Rac1, Cdc42, and RhoA in PANC-1 shNT and shECT2 knockdown cells; *n* = 3; results expressed as % shNT and represent the mean ± SEM. (*) p < 0.05 compared to shNT and (**) *p* < 0.05 compared to vector in (**B**–**G**).

Rho GTPases are well characterized for their role in PDAC transformation and GEFs represent a major mechanism by which the Rho GTPases are aberrantly activated in cancer [30, 31]. ECT2 is a member of the DBL family of GEFs with specificity for Rac1, RhoA and Cdc42 activation [27]. Introducing E428A and N608A mutations within the DBL homology domain abolishes ECT2 GEF activity (DHmut-ECT2) (Fig. 6A). To assess the importance of ECT2 GEF activity in PDAC cell transformation, we expressed ECT2 shRNA resistant cDNAs of HA-tagged WT-ECT2 or DHmut-ECT2 in shECT2 cells, reconstituting ECT2 expression to endogenous levels (Fig. 6A). Unlike WT-ECT2, DHmut-ECT2 failed to rescue soft agar growth, invasion, or clonal expansion (Figs. 6B–E and S6A–E), indicating that ECT2 GEF activity is essential for PDAC oncogenic functions.

### ECT2 is required for Rac1 and RhoA activation in PDAC cells

Since we observed that ECT2 GEF activity is required for ECT2-mediated transformed growth and invasion in PDAC cells, we next assessed if ECT2 expression associates with the activity of the Rho-GTPases in PDAC cells. We performed pull-down assays to assess the levels of active, GTP-bound Rac1, RhoA, and Cdc42 in shNT control and shECT2 PDAC cells. These assays revealed a reduction in the active forms of Rac1 and RhoA in shECT2 cells, whereas the levels of active Cdc42 remained unchanged (Fig. 6F, G). Our results suggest that ECT2 activation of Rac1 and RhoA is required for the transformed phenotype of PDAC cells.

### ECT2 drives MEK/ERK signaling in PDAC cells

**Fig. 7:**
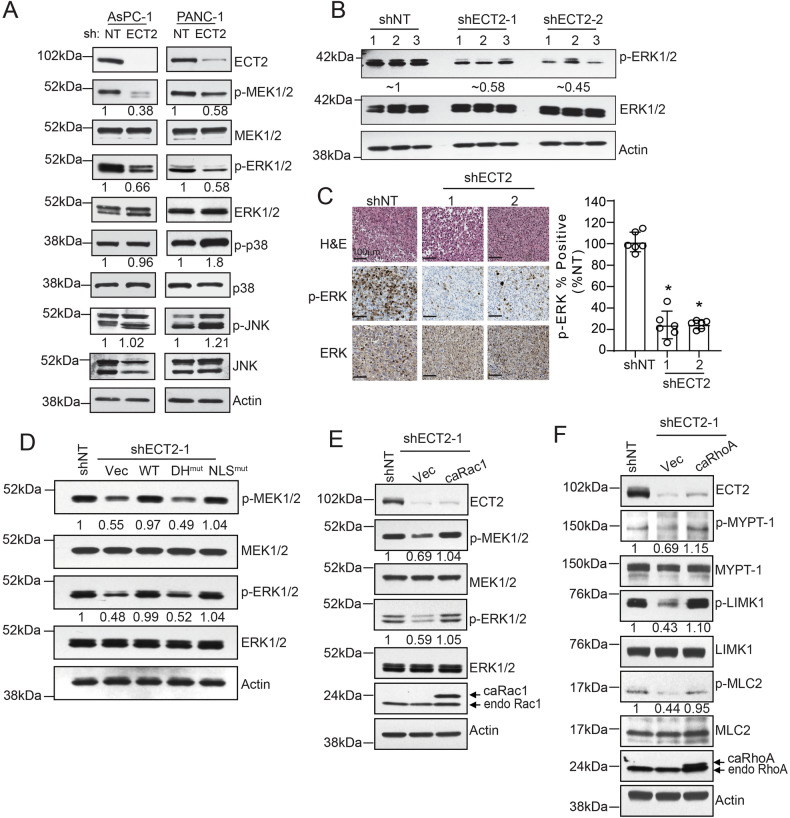
ECT2 mediates MEK/ERK and ROCK signaling in PDAC cells. **A** Immunoblot analysis of phosphorylated and total MEK1/2, ERK1/2, p38, and JNK in AsPC-1 and PANC-1 shNT and shECT2 cells. **B** Immunoblot analysis of phosphorylated and total ERK1/2 in PANC-1 shNT and shECT2 subcutaneous tumors. **C** Representative photomicrographs of hematoxylin and eosin (H&E) and phosphorylated and total ERK1/2 IHC in PANC-1 shNT and shECT2 subcutaneous tumors. Quantitated results expressed as % shNT and represent the mean ± SEM. **D** Immunoblot analysis of phosphorylated and total MEK1/2, ERK1/2 levels in vector (Vec) or WT, DH^mut^ or NLS^mut^ ECT2 expressing shECT2 knockdown cells. **E** Immunoblot analysis of the effects of expression of constitutively active Rac1 (caRac1V12) in AspC-1 shECT2 knockdown cells on the levels phosphorylated and total MEK1/2 and ERK1/2. **F** Immunoblot analysis of the effects of expression of constitutively active RhoA (caRhoAV17) in AsPC-1 shECT2 knockdown cells on the levels phosphorylated and total LIMK, MYPT-1 and MLC2. (*) *p* < 0.05 compared to NT. B-actin served as loading control and phospho-proteins were normalized to the corresponding total protein, and expressed relative to NT.

Our data indicate that ECT2 stimulates PDAC cell proliferation to promote PDAC tumor growth. The MAPK pathway components ERK, p38 and JNK have been shown to play critical roles in cell proliferation [32, 33] and aberrant activation of the MEK/ERK signaling pathway, in particular, is a hallmark of PDAC. Rac1 activation of its effector, p21-activated kinase 1 (PAK1), is reported to stimulate PAK1-mediated phosphorylation of MEK at S298, which has been shown to be necessary for efficient activation of MEK and subsequent MAPK activation [34, 35]. Given that ECT2 knockdown decreases Rac1 activation, we examined the effects of ECT2 knockdown on MEK S298 phosphorylation status. We found that shECT2 knockdown cells exhibited significantly lower levels of phospho-298-MEK1,2 compared to shNT cells, with total MEK unchanged (Figs. 7A and S7A). Consistent with the decrease in MEK phosphorylation, we also observed a decrease in MEK phosphorylated ERK1,2 upon knockdown of ECT2 with no impact on the levels of total ERK1,2 (Figs. 7A and S7A). The Rho-GTPases have also been linked to the activation of JNK and p38 [36]. Densitometric analysis confirmed suppression of MEK/ERK signaling following ECT2 depletion and revealed an increase in phospho-p38 and phospho-JNK levels in shPANC-1 cells (Figs. 7A and S7A), suggesting that a stress-associated MAPK response may be activated following disruption of ECT2 signaling in some PDAC cells. Immunoblot and IHC analysis showed a marked decrease in phospho-ERK levels between shNT and shECT2 PANC-1 and MiaPaCa-2 xenograft tumors (Figs. 7B, C and S7B), suggesting that ECT2 stimulates ERK activation in PDAC tumor growth.

To further investigate the role of ECT2 in MEK/ERK activation, we assessed the requirement for ECT2 GEF activity and cytoplasmic/nuclear localization. WT-ECT2 and NLSmut-ECT2, but not DHmut-ECT2, restored phospho-MEK and -ERK, indicating that cytoplasmic ECT2 is sufficient for MEK/ERK activation (Figs. 7C, D and S7B). In addition, the expression of a constitutively active Rac1 (caRac1) allele in PANC-1 and AsPC-1 shECT2 cells was able to reconstitute phospho-MEK1,2 and -ERK1,2 (Figs. 7E and S7C), further supporting that MEK/ERK is an effector of ECT2-Rac1 signaling in PDAC cells.

### ECT2 mediates RhoA/ROCK signaling

RhoA GTPase activity assays show a decrease in GTP-RhoA in shECT2 PDAC cells (Fig. 6F, G)*.* GTP-RhoA activates ROCK which in turn phosphorylate downstream effectors LIMK, MYPT1 and MLC2 resulting in enhanced actomyosin contractility that facilitates cell migration and invasion [37, 38]. Immunoblot analysis revealed that ECT2 knockdown decreased phosphorylated LIMK, MYPT1 and MLC2 levels (Figs. 7F and S7D). The expression of a constitutively active RhoA (caRhoA) in shECT2 cells rescued phospho-LIMK, -MYPT1 and -MLC2 levels (Figs. 7F and S7D). RhoA signaling has been implicated in the activation of MEK/ERK signaling [39, 40]. However, expression of caRhoA in shECT2 cells had little effect on the expression of phospho-MEK1,2 and -ERK1,2 (Fi. S7E). Likewise, expression of caRac1 in shECT2 cells had no effect on phospho-LIMK, -MYPT1, and -MLC2 (Fig. S7F) levels. These data suggest that ECT2-mediated activation of Rac1 and RhoA in PDAC cells activate distinct signaling pathways.

### High ECT2 expression associates with poor survival and correlates with Rho GTPase and MAPK/ERK signatures in human primary PDAC tumors

**Fig. 8:**
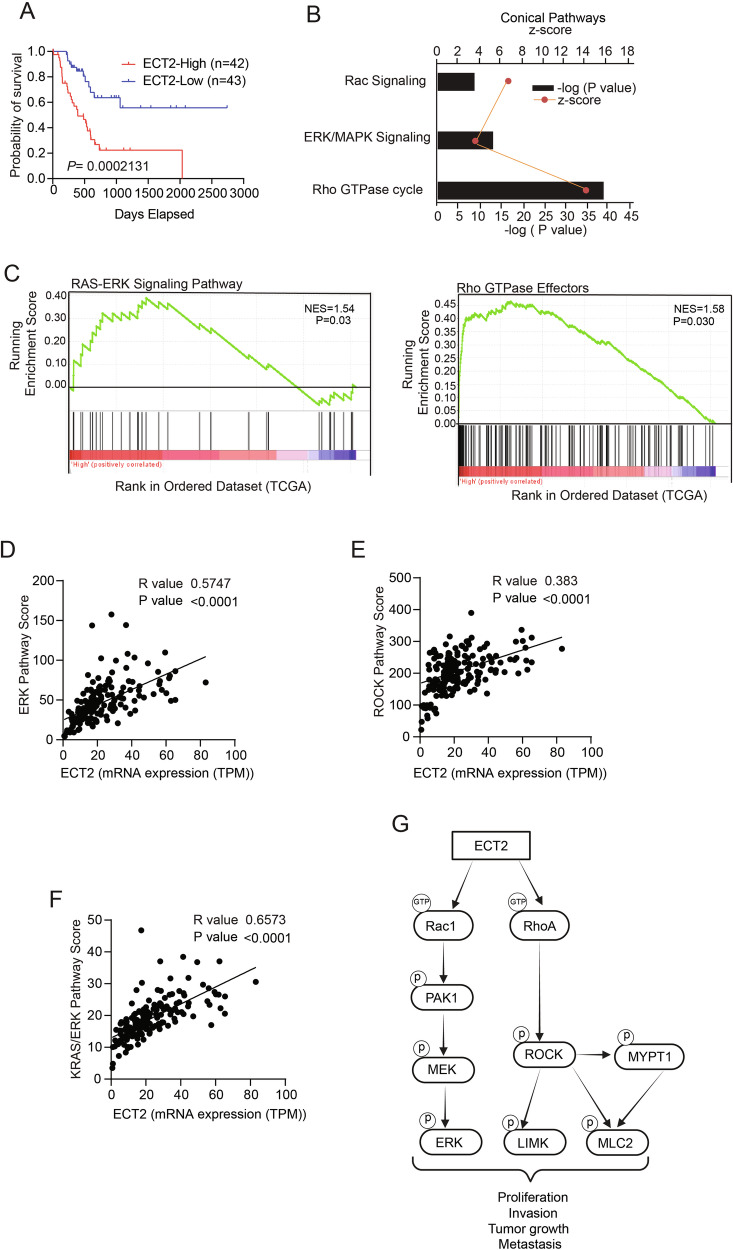
High ECT2 expression associates with poor survival and MAPK/ERK and RHO GTPase signaling in human primary PDAC tumors. **A** Kaplan–Meier analysis of overall survival for TCGA PDAC patients with the top versus the bottom quartile of ECT2 expressing tumors (top, *n* = 42; bottom, *n* = 43). **B** Ingenuity pathway analysis of significant differentially expressed genes (*P*-value < 0.05) between the top versus the bottom quartile of ECT2 expressing tumors revealed enrichment of genes associated with RAC, ERK/MAPK and RHO GTPase cycle signaling pathways. Bars represent the (log) *P*-values of the significance of the pathway and points on the line graph indicate z scores. **C** Gene Set Enrichment Analysis (GSEA) of differentially expressed genes in ECT2-high versus ECT2- low PDAC tumors showing RAS-ERK and RHO GTPases Effectors are significantly altered between groups. **D**–**F** Scatterplots showing the correlation between ECT2 expression and ERK, ROCK and KRAS/ERK pathway signature scores in TCGA PDAC dataset (*n* = 179). **G** Proposed model of ECT2 mediated regulation of Rac1–MEK/ERK and RhoA–ROCK signaling pathways in PDAC cells.

We observed elevated expression of ECT2 in primary PDAC tumors (Fig. 1). To assess the clinical relevance of ECT2 expression and signaling in human PDAC tumors, we first assessed the relationship between ECT2 expression and patient survival. In the TCGA PAAD cohort, patients with the highest quartile of ECT2 expression had significantly shorter median survival (16.7 vs 33.3 months) than those in the lowest quartile (Fig. 8A). Differential gene expression analysis followed by Ingenuity Pathway Analysis (IPA) revealed enrichment of Rac, Rho-GTPase cycle and ERK/MAPK pathways in ECT2-high tumors (Fig. 8B). Gene set enrichment analysis (GSEA) also showed enrichment of signatures corresponding to RAS/ERK and Rho-GTPase effector signaling in ECT2-high PDAC tumors (Fig. 8C). Interestingly, ERK and ROCK pathway signature scores correlated significantly stronger with ECT2 than with other PDAC linked Rho-GEFs including VAV1, TIAM1, DOCK8 (Figs. 8D, E and S8A, B). These data indicate a selective involvement of ECT2 in PDAC MEK/ERK and ROCK signaling.

The transforming potential of the Rho GTPases has been attributed to their ability to cause changes in actin cytoskeletal organization, promote cell cycle progression and alter gene expression [21, 41–43]. Recently, the contribution of ERK/MAPK signaling in driving KRAS-regulated gene transcription was determined in PDAC cells [21]. A KRAS-ERK UP gene signature was derived based on differential expressed genes from ERK inhibitor, KRAS siRNA, and KRAS inhibitor treated versus control cells. We found a strong association between the KRAS-ERK UP gene signature and ECT2 expression in PDAC primary tumors (Fig. 8F), supporting a role for ECT2 in reinforcing the KRAS-ERK–driven transcriptional program that contributes to PDAC pathogenesis. These results are consistent with our data demonstrating that ECT2 stimulates MEK/ERK and ROCK signaling in PDAC cells (Fig. 8G).

### Targeting of ECT2-Rac1 signaling enhances PDAC cell sensitivity to MEK inhibition

**Fig. 9:**
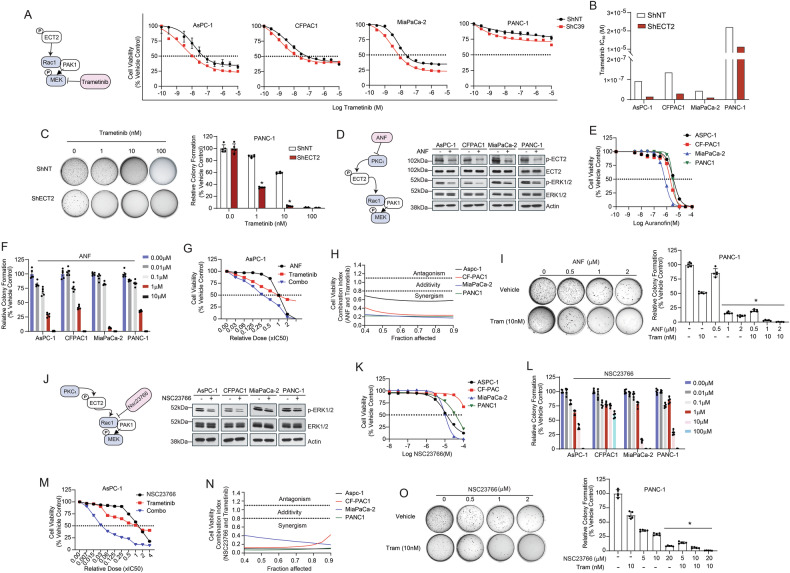
Targeting of ECT2-Rac1 signaling enhances PDAC cell sensitivity to MEK inhibition. **A** Dose–response curves showing cell viability of AsPC-1, CFPAC-1, MiaPaCa-2, and PANC-1 cells expressing shNT or shECT2 following treatment with increasing concentrations of Trametinib; *n* = 5. **B** Trametinib IC50 values in PDAC cell lines expressing shNT or shECT2. **C** Soft agar colony formation of PANC-1 cells expressing shNT or shECT2 treated with increasing concentrations of Trametinib; *n* = 5 * *p* < 0.05 relative to vehicle treated control. **D** Schematic representation of PKCι inhibition by ANF within the PKCι–ECT2–Rac1–PAK–MEK/ERK signaling pathway and immunoblot analysis of AsPC-1, CFPAC-1, MiaPaCa-2, and PANC-1 cells treated with ANF showing p-ECT2, ECT2, p-ERK1/2, and ERK1/2 expression. Dose–response curves showing cell viability of PDAC cell lines (**E**) and quantification of relative colony formation (**F**) following treatment with increasing concentrations of ANF; *n* = 5; * *p* < 0.05 relative to vehicle treated control. **G** Dose–response analysis of ANF and Trametinib alone or in combination in AsPC-1 cells; *n* = 5. **H** Combination index (CI) analysis of ANF and Trametinib in PDAC cells. CI < 0.9 indicates synergism, CI between 0.90 and 1.20  indicates additivity, and CI > 1.2 indicates antagonism. **I** Representative images and quantitation of colony formation of PANC-1 cells treated with ANF alone, Trametinib alone, or the combination of ANF and Trametinib; *n* = 5; * *p* < 0.05 relative to vehicle treated control. ** *p* < 0.05 relative to Trametinib alone treatment. **J** Immunoblot analysis of PDAC cell lines treated with the Rac1 inhibitor NSC23766 showing total and phosphorylated ERK1/2 status. Dose–response curves showing cell viability of PDAC cell lines (**K**) and quantification of relative colony formation (**L**) following treatment with increasing concentrations of with NSC23766; *n* = 5; * *p* < 0.05 relative to vehicle treated control. **M** Relative dose–response curves showing the combined effects of NSC23766 and Trametinib treatment in AsPC-1 cells; *n* = 5. **N** CI analysis showing the interaction between NSC23766 and Trametinib treatment on the viability of PDAC cells. **O** Representative images and quantitation of colony formation of PANC-1 cells treated with NSC23766 alone, Trametinib alone, or the combination of NSC23766 and Trametinib. * p < 0.05 relative to vehicle treated control. ** p < 0.05 relative to Trametinib alone treatment. For immunoblots, B-actin served as loading control and phospho-proteins were normalized to the corresponding total protein and expressed relative to vehicle treated cells.

Since ECT2 activates Rac1-dependent MEK/ERK signaling in PDAC cells, we next assessed whether targeting the ECT2-Rac1 signaling axis alters the response of PDAC cells to MEK inhibition. We first examined the effect of ECT2 knockdown on sensitivity to the MEK inhibitor Trametinib in a panel of PDAC cell lines including two Trametinib-resistant PDAC cell lines (CFPAC1 and PANC-1) and two Trametinib-sensitive PDAC cell lines (ASPC-1 and MiaPaCa-2). Knockdown of ECT2 significantly increased sensitivity to Trametinib, reducing the concentration required to suppress PDAC cell viability (Fig. 9A, B). Consistent with these findings, ECT2 depletion also suppressed clonogenic growth following Trametinib treatment, indicating that ECT2 knockdown leads to sustained suppression of transformed growth under MEK inhibition (Figs. 9C and S9A). These results indicate that targeting ECT2 signaling enhances the response of PDAC cells to MEK inhibition.

There are currently no small molecule inhibitors that directly target ECT2. To pharmacologically interrogate ECT2-dependent signaling in PDAC cells, we targeted ECT2 signaling both upstream and downstream of ECT2. We previously demonstrated that PKCι phosphorylates ECT2 on Thr328 to regulate ECT2-dependent Rho GTPase activation and oncogenic function [44]. The PKCι inhibitor Auranofin (ANF) blocks PKCι-mediated phosphorylation of ECT2 at Thr328 and suppresses transformed growth of NSCLC cells [13]. ANF treatment reduced ECT2 and ERK phosphorylation across PDAC cell lines (Fig. 9D), consistent with inhibition of ECT2-dependent MAPK signaling. ANF treatment produced dose-dependent inhibition of PDAC cell viability (Fig. 9E) and suppressed anchorage-independent transformed growth (Figs. 9F and S9A), indicating that pharmacologic targeting of the PKCι-ECT2 axis inhibits PDAC cell growth. Interestingly, combined treatment with ANF and Trametinib produced greater inhibition of PDAC cell viability than either agent alone (Figs. 9G and S9C). Combination index analysis demonstrated a synergistic interaction between PKCι-ECT2 pathway and MEK inhibition across multiple PDAC cell lines (Fig. 9H). Similar effects were observed in anchorage-independent growth assays (Figs. 9I and S9D),

ECT2 activates Rac1 to stimulate MEK/ERK, therefore we tested whether targeting Rac1 phenocopies the effects of ECT2 suppression. Treatment of PDAC cells with the Rac1 inhibitor NSC23766 reduced ERK phosphorylation (Fig. 9J) and produced dose-dependent inhibition of PDAC cell viability (Fig. 9K) anchorage-independent growth (Figs. 9L and S9E). Rac1 inhibition also enhanced sensitivity to Trametinib and demonstrated synergistic activity when combined with MEK inhibition (Figs. 9M–P and S9F, G). ECT2 can also activate RhoA signaling. We therefore tested whether inhibition of the downstream RhoA-ROCK pathway affects PDAC cell growth using the ROCK inhibitor Y-27632. In contrast to Rac1 inhibition, ROCK inhibition produced relatively modest effects on PDAC cell viability (Fig. S9H), indicating that Rac1 inhibition produced stronger growth inhibitory effects than inhibition of the RhoA–ROCK pathway. Together, these findings indicate that suppression of upstream signals that activate ECT2 or the downstream ECT2 effector Rac1 enhances the response of PDAC cells to MEK inhibition.

## Discussion

PDAC is one of the deadliest human malignancies due to its aggressive nature, late-stage diagnosis, and limited therapeutic options [45]. Mutations in KRAS are nearly ubiquitous in PDAC and are known to drive tumor initiation and progression by activating key downstream pathways, including the RAF-MEK-ERK cascade and the Rho family of GTPases [3, 8]. Although these signaling axes are well-characterized, the molecular mediators that coordinate and amplify their effects remain incompletely defined. In this study, we identify the Rho-GEF ECT2 as a critical effector that links KRAS signaling to both the Rho GTPase and MEK/ERK pathways in PDAC.

Our findings demonstrate that ECT2 is overexpressed early in PDAC, with elevated expression detectable in PanIN lesions and maintained in invasive carcinomas (Fig. 1 and Supplementary Fig. 1), suggesting a role for ECT2 in both PDAC tumor initiation and maintenance. Our findings align with prior reports documenting aberrant ECT2 overexpression in other tumor types, including lung, ovarian, colorectal, and liver cancers [13–16], suggesting that ECT2 upregulation may represent a common feature across cancers. ECT2 overexpression likely results from multiple mechanisms, including copy number gains at the *ECT2* locus, which are present in a subset of PDAC tumors (Fig. 1B, C). In addition, transcriptional regulation appears to be a key driver of ECT2 expression. One potential upstream driver is YAP1, a transcriptional coactivator frequently activated in PDAC and known to cooperate with mutant KRAS during early tumorigenesis [46]. YAP1 has been shown to transcriptionally activate ECT2 in KRAS-mutant PDAC cells [18, 47]. Furthermore, YAP1-driven ECT2 expression has been implicated as a mechanism of resistance to MEK inhibition, helping tumor cells bypass ERK pathway blockade by activating compensatory Rho GTPase signaling [47]. We show that ECT2 expression correlates with KRAS-ERK gene signatures in PDAC tumors (Fig. 8C, D, F), and ECT2 is required for MEK/ERK activation. These findings support a model in which KRAS signaling induces ECT2 through transcription factors such as YAP1, establishing a feedforward loop wherein ECT2 activation of Rho GTPases sustains ERK phosphorylation. Taken together, our results position ECT2 as both a KRAS-responsive gene and a critical amplifier of its oncogenic signaling, with potential implications for therapeutic resistance and progression in PDAC.

The Rho family of small GTPases, including Rac1, Cdc42, and RhoA, are key regulators of cytoskeletal dynamics, cell polarity, and migration, and have been increasingly recognized as critical effectors of mutant KRAS-driven tumorigenesis. In pancreatic and lung cancer models genetic deletion of Rac1 impairs tumor initiation and progression, demonstrating that Rac1 is a critical downstream mediator of oncogenic KRAS [48, 49]. Similarly, RhoA has been shown to cooperate with KRAS to promote oncogenic phenotypes, including actomyosin contractility and invasion, often through activation of ROCK1/2 signaling [50]. Mechanistically, KRAS engages these GTPases either directly via PI3K and PAK signaling cascades or indirectly through upregulation of specific Rho-GEFs, which activate Rho proteins by facilitating GDP-GTP exchange [8]. In this study, we found that ECT2-mediated PDAC transformed growth and invasion is dependent on its GEF activity. Specifically, we find that ECT2 activates both RhoA and Rac1 but has no appreciable effect on Cdc42 activation in PDAC cells (Fig. 6). Furthermore, our study provides insight into the relative contribution of ECT2 signaling through these GTPases to regulate PDAC phenotypes. Our data is consistent with previous reports of Rac1 activation of PAK1, which in turn primes MEK for full activation [34, 35] and subsequent ERK 1,2 signaling. In parallel, we demonstrate that ECT2 activates the RhoA-ROCK axis, as shown by reduced phosphorylation of LIMK, MLC2 and MYPT1, following ECT2 knockdown, and restoration of these proteins by constitutively active RhoA. Interestingly, Rac1 and RhoA signaling downstream of ECT2 appear to operate independently, as active Rac1 did not restore ROCK pathway effectors, nor did active RhoA restore MEK 1, 2 activation. The dual regulation of Rac1-MEK/ERK and RhoA-ROCK by ECT2 highlights its central role in coordinating transformed growth and invasion in PDAC cells. Targeting ECT2 may therefore simultaneously impair tumor cell growth and motility, providing a potentially more comprehensive therapeutic approach than inhibiting either pathway alone. These data support a model in which ECT2 functions as a central hub, coordinating parallel signaling axes via distinct GTPases (Fig. 8G).

In non-transformed cells, ECT2 exhibits dynamic distribution during the cell cycle, localizing to the nucleus in interphase and translocating to the mitotic spindle and cleavage furrow during mitosis to regulate cytokinesis [27]. We noted altered subcellular distribution of ECT2 in PDAC cells with ECT2 localizing in both the nuclear and cytoplasmic compartments of PDAC cells during interphase (Figs. 5A and S5A). This cytoplasmic mislocalization of ECT2 could lead to aberrant signaling by disrupting its spatial regulation of Rho GTPase activity, particularly Rac1 and RhoA, both of which are known to influence tumor cell motility and proliferative signaling [8]. Indeed, cytoplasm-restricted ECT2 is sufficient to rescue ERK activation and partially restore transformed phenotypes, suggesting that cytoplasmic ECT2 supports MEK/ERK pathway activation. However, full rescue requires nuclear ECT2, consistent with previous findings showing that nuclear ECT2 regulates additional tumor-promoting processes necessary for ECT2-mediated transformation [13, 16]. In this regard, 45S rRNA, which is critical for supporting the high protein biosynthesis demands of rapidly proliferating cancer cells and is regulated by nuclear ECT2 in lung and colon cancer [13, 16], is reduced in PDAC shECT2 cells (Fig. 5I). 45S rRNA levels in PDAC shECT2 cells could be rescued by wild-type but not cytoplasm-restricted ECT2 (Fig. 5J), reinforcing the idea that ECT2’s nuclear function supports protein synthesis and proliferation. These findings underscore the importance of ECT2’s dual localization in PDAC biology and suggest that ECT2 oncogenic functions are spatially compartmentalized and required cooperatively for PDAC growth and invasion.

Interestingly, the oncogenic functions of ECT2 in PDAC appear to be context-dependent. In non-transformed cells, ECT2 regulates cytokinesis by activating RhoA to promote assembly of the actomyosin contractile ring and cleavage furrow formation during cell division. Consistent with this role, ECT2 depletion in normal HPDE cells impaired proliferation, increased population doubling time, and induced multinucleation under 2D anchorage dependent conditions, indicative of cytokinesis defects (Figs. 4A, B and S4A). In contrast, ECT2 knockdown in PDAC cells did not induce multinucleation or alter population doubling time under adherent growth conditions, suggesting that PDAC cells have uncoupled cell growth from ECT2-dependent cytokinesis. Similar findings have been reported in several cancer models, including lung, ovarian, and colon cancer cells. One possible explanation is that residual ECT2 expression in knockdown cells may remain above a critical threshold required for completion of cytokinesis. Alternatively, PDAC cells may utilize mechanisms that bypass the requirement for ECT2 during cytokinesis, as has been described in fibrosarcoma cells, where an alternative ECT2-independent cytokinesis mechanism enables cell division despite ECT2 depletion [51]. Consistent with this model, ECT2 depletion markedly impaired anchorage-independent/transformed growth in soft agar and spheroid assays, whereas PDAC cells retained proliferative capacity under standard adherent culture conditions (Figs. 2, 4A, B and S4A). Our data indicate that ECT2 promotes transformed growth in PDAC cells through activation of Rac1-dependent MEK/ERK signaling, highlighting a tumor-specific function that sustains oncogenic signaling pathways and is mechanistically distinct from its canonical role in cytokinesis.

The significance of ECT2 overexpression in PDAC is underscored by its strong association with reduced patient survival and enrichment of MAPK/ERK and Rho GTPase pathway gene signatures in human PDAC datasets. Among the various Rho-GEFs implicated in PDAC, including VAV1, TIAM1, and DOCK8 [9–11], ECT2 appears to have a particularly stronger association with MEK/ERK and ROCK signaling activity (Figs. 8 and S8). This specificity may reflect a unique function for ECT2 in KRAS-driven signaling and suggests that ECT2 could represent an attractive target for therapeutic intervention. Consistent with this concept, pharmacologic inhibition of ECT2 signaling upstream with the PKCι inhibitor ANF reduced phosphorylation of ECT2 and ERK activation in PDAC cells, indicating that inhibition of ECT2 activation attenuates downstream MAPK signaling. Similarly, inhibition of Rac1 using NSC23766 reduced ERK phosphorylation and suppressed PDAC cell growth, consistent with Rac1 functioning as a key downstream effector of oncogenic ECT2 signaling. Importantly, genetic depletion of ECT2 or pharmacologic targeting of ECT2 signaling with Auranofin or NSC23766 increased the sensitivity of PDAC cells to MEK inhibition across both Trametinib-sensitive and Trametinib-resistant PDAC cell lines (Fig. 9). Given the limited clinical utility of direct MEK inhibition in PDAC due to toxicity and compensatory signaling loops, targeting upstream regulators such as ECT2 may provide a more effective strategy to attenuate MAPK pathway activity. Inhibiting ECT2 might also allow for dose-sparing combinations with MEK inhibitors, thereby enhancing efficacy while reducing toxicity. Together, these results support the concept that pharmacologic disruption of the ECT2-Rac1 signaling axis could represent a rational strategy to enhance responses to MAPK pathway inhibitors in PDAC.

The in vivo pre-clinical models used in this study provide strong evidence that ECT2 is required for PDAC tumor growth, vascularization, and metastatic dissemination. These models primarily assess tumor cell intrinsic functions and do not fully capture the complex immune and stromal interactions present in autochthonous pancreatic tumors. Future studies using immunocompetent genetically engineered mouse models incorporating conditional ECT2 loss or gain of function in KRAS-driven PDAC will further clarify the role of ECT2 in pancreatic tumor initiation, progression and tumor microenvironment interactions. In addition, while our biochemical and functional studies establish distinct nuclear and cytoplasmic pools of ECT2 that cooperate to sustain PDAC transformation, future live cell imaging approaches may provide additional insight into the dynamic spatial regulation of ECT2 signaling. We observed a profound suppression of transformed growth following ECT2 depletion. Interestingly, ECT2 depletion increased phosphorylation of p38 and JNK in PANC-1 cells (Fig. 7A). Since p38 and JNK are commonly activated in response to cellular stress, this finding suggests activation of a compensatory stress signaling response to ECT2 loss. Future genome-scale approaches, such as RNA sequencing or proteomic profiling may provide additional further insight into adaptive responses to ECT2 inhibition that may develop in PDAC cells. Our functional studies identify Rac1 and RhoA as principal downstream effectors of oncogenic ECT2 signaling in PDAC, and the enrichment of Rho GTPase and MEK/ERK pathway signatures in ECT2 high patient tumors supports the clinical relevance of this signaling axis. Proteomic analyses may identify additional ECT2-associated signaling complexes that contribute to oncogenic signaling in PDAC and represent potential therapeutic vulnerabilities.

In conclusion, this study identifies an important role for ECT2 in PDAC pathogenesis, functioning at the intersection of Rho GTPase activation and MAPK signaling. Overexpression early in tumorigenesis, dual subcellular roles that support PDAC cell invasion and tumor growth, and a strong prognostic association position ECT2 as a compelling candidate for targeted therapy.

## Supplementary information

Supplemental Figure 1

Supplemental Figure 2

Supplemental Figure 3

Supplemental Figure 4

Supplemental Figure 5

Supplemental Figure 6

Supplemental Figure 7

Supplemental Figure 8

Supplemental Figure 9

Supplemental Table 1

Supplemental Table 2

Supplemental Table 3

Supplementary figure legends

## Data Availability

All data supporting the findings of this study are included within the article and its supplementary materials. Further information and requests should be directed to the lead contact.
