# Supplementary figures and images for: Early induction of the Rho-GEF ECT2 drives MEK/ERK oncogenic signaling in pancreatic ductal adenocarcinoma
Source: Oncogene. 2026 Jun 19;45(30):3053–68. doi: 10.1038/s41388-026-03860-3 (PMC13384901; doi:10.1038/s41388-026-03860-3)

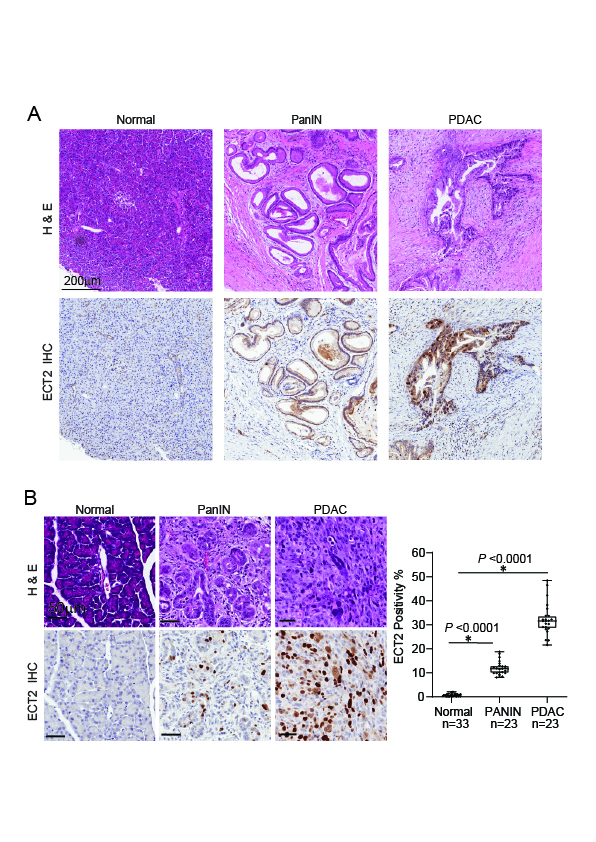

Supplement: Supplementary file 1 — Supplemental Figure 1 [file 41388_2026_3860_MOESM1_ESM.jpg]

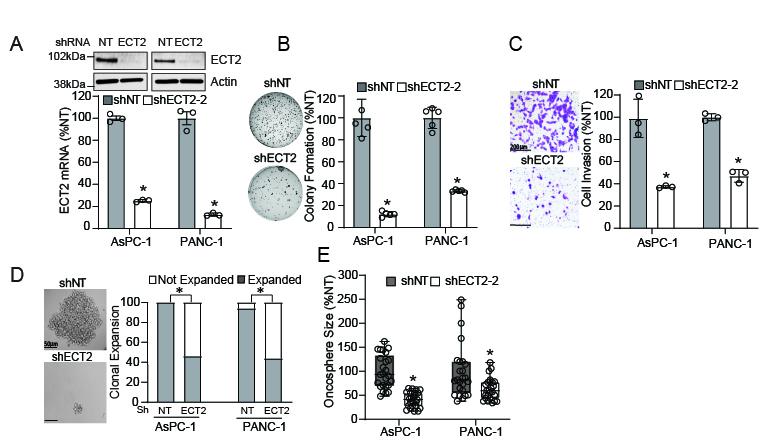

Supplement: Supplementary file 2 — Supplemental Figure 2 [file 41388_2026_3860_MOESM2_ESM.jpg]

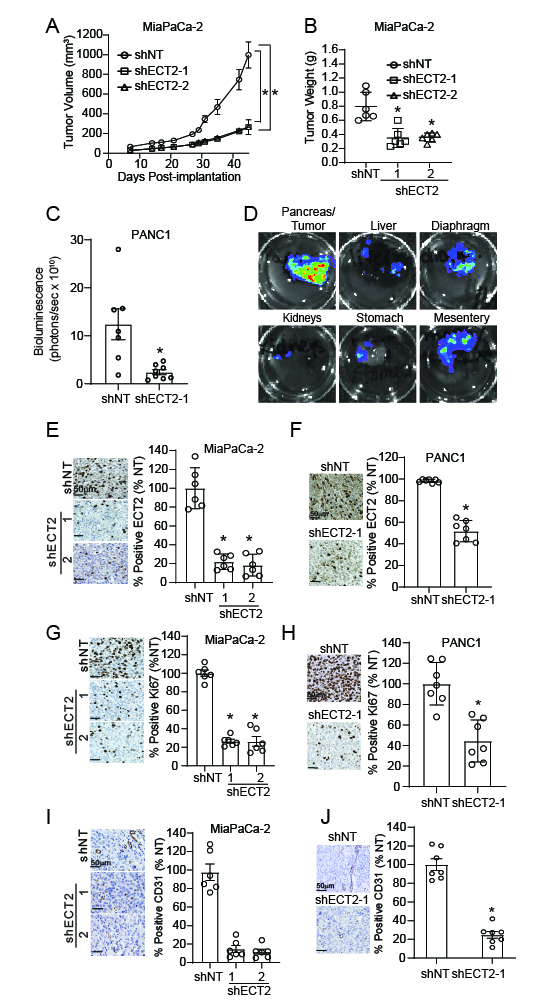

Supplement: Supplementary file 3 — Supplemental Figure 3 [file 41388_2026_3860_MOESM3_ESM.jpg]

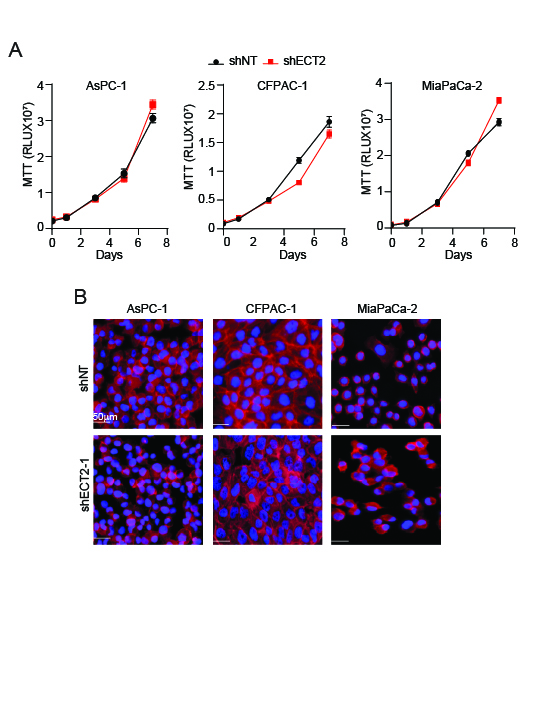

Supplement: Supplementary file 4 — Supplemental Figure 4 [file 41388_2026_3860_MOESM4_ESM.jpg]

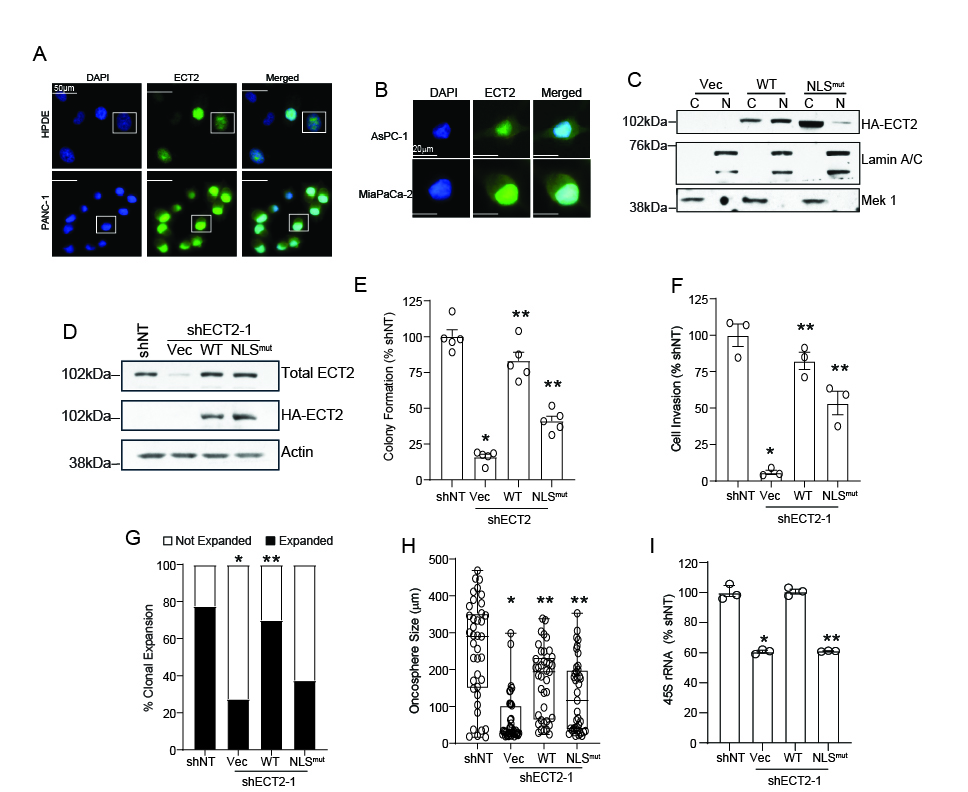

Supplement: Supplementary file 5 — Supplemental Figure 5 [file 41388_2026_3860_MOESM5_ESM.jpg]

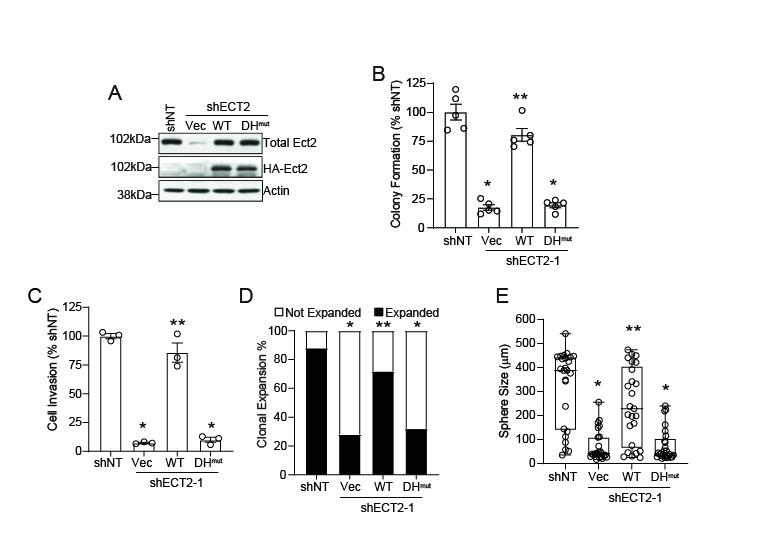

Supplement: Supplementary file 6 — Supplemental Figure 6 [file 41388_2026_3860_MOESM6_ESM.jpg]

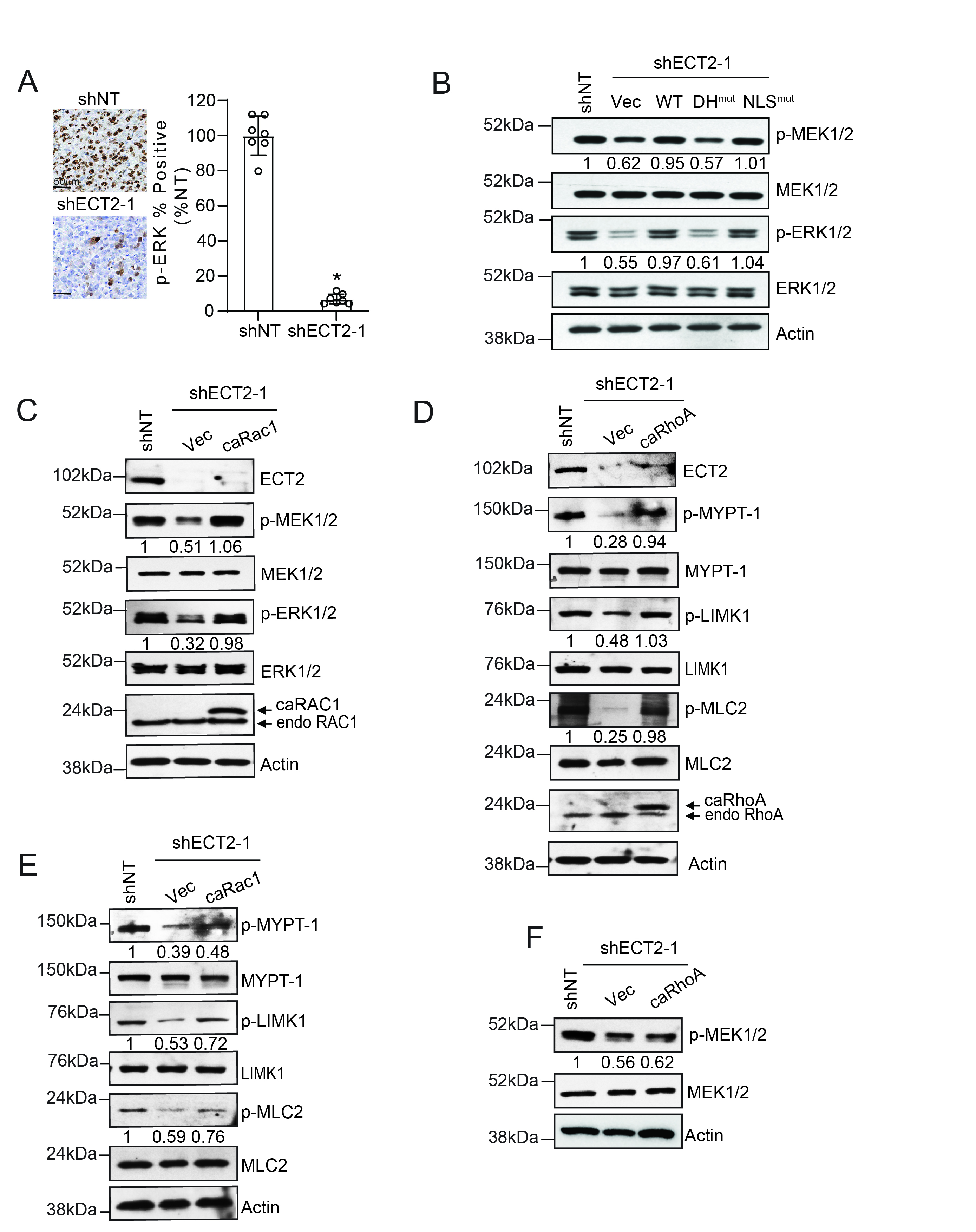

Supplement: Supplementary file 7 — Supplemental Figure 7 [file 41388_2026_3860_MOESM7_ESM.jpg]

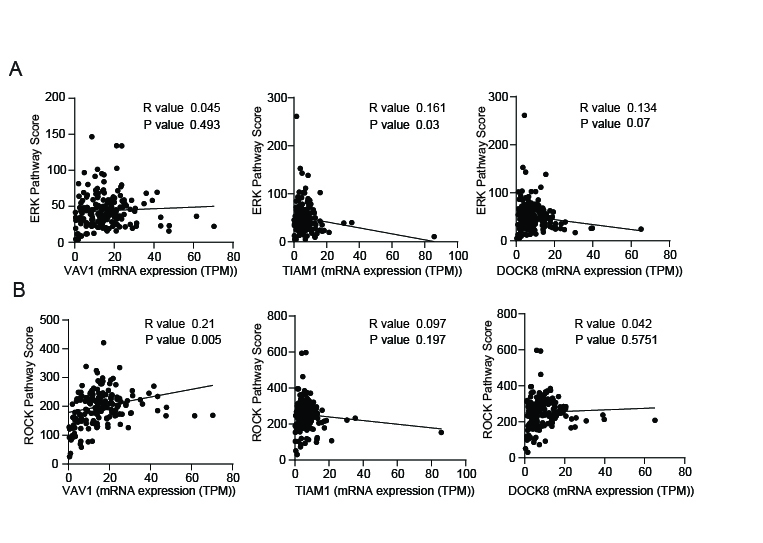

Supplement: Supplementary file 8 — Supplemental Figure 8 [file 41388_2026_3860_MOESM8_ESM.jpg]

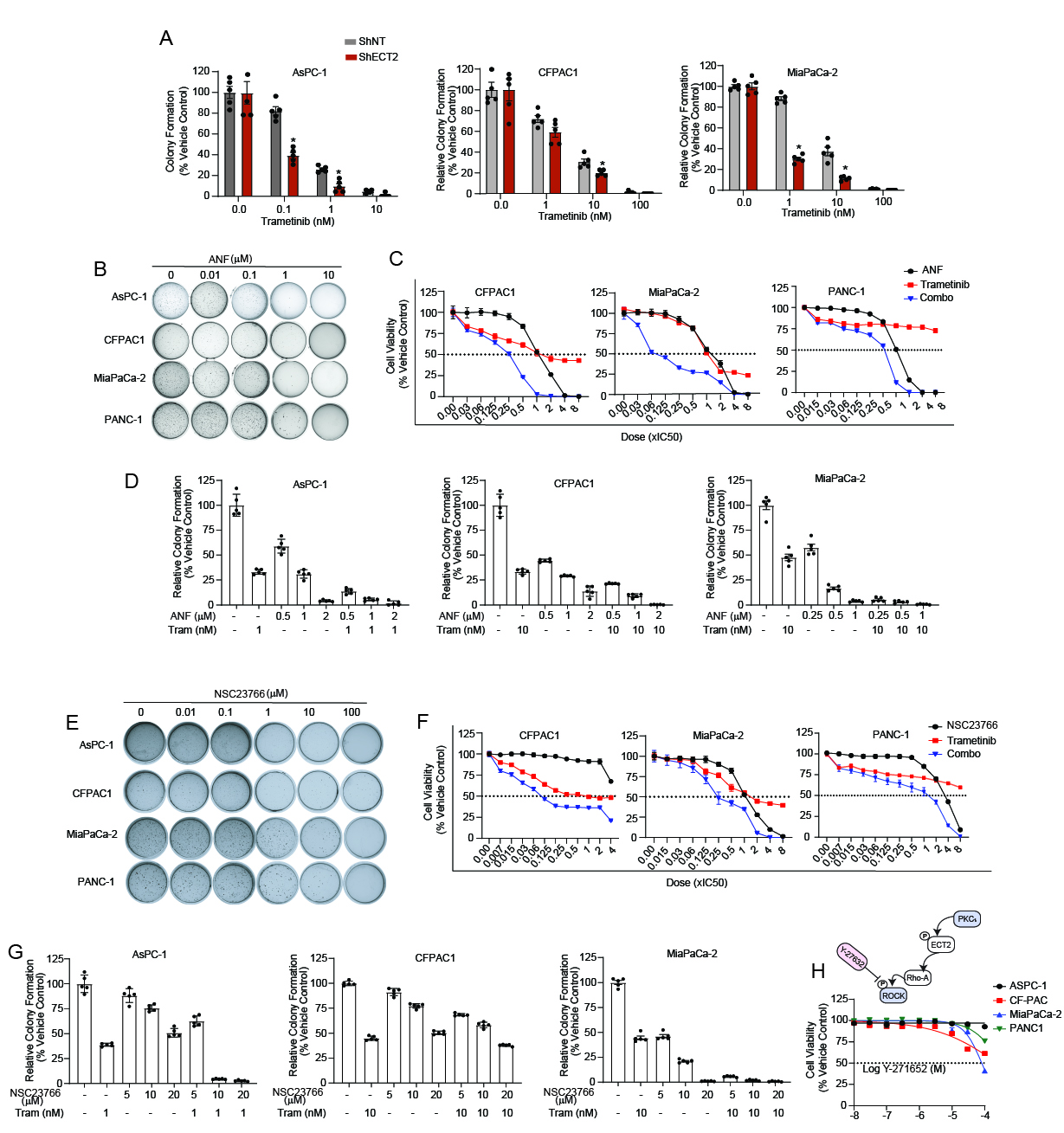

Supplement: Supplementary file 9 — Supplemental Figure 9 [file 41388_2026_3860_MOESM9_ESM.jpg]
