# Supplementary material for: Early induction of the Rho-GEF ECT2 drives MEK/ERK oncogenic signaling in pancreatic ductal adenocarcinoma
Source: Oncogene. 2026 Jun 19;45(30):3053–68. doi: 10.1038/s41388-026-03860-3 (PMC13384901; doi:10.1038/s41388-026-03860-3)
Supplement: Supplementary file 13 — Supplementary figure legends [file 41388_2026_3860_MOESM13_ESM.docx]

**Supplementary Figures**

**Figure S1. ECT2 overexpression occurs early in PDAC tumorigenesis.** **A.** Extended field of view of H&E and ECT2 IHC in human non-tumor pancreas, PanIN and PDAC tissue. **B.** Representative H&E and ECT2 IHC images and plot of Ect2 histoscore in mouse non-tumor pancreas, PanIN and PDAC in the Kras^G12D/+^;LSL-Trp53^R172H/+^;Pdx-Cre mouse model; n=33 normal; 23 PanIN; 23 PDAC lesions from 9 mice.

**Figure S2. ECT2 is required for PDAC transformed phenotypes.** **A** qPCR and immunoblot analysis of ECT2 mRNA and protein abundance in AsPC-1 and PANC-1 shECT2 knockdown cells generated with a second independent ECT2 shRNA (shECT2-2); n=3. **B** Representative photomicrographs and quantitation of soft agar colonies formed by PDAC shNT and shECT2 cells; n=5. **C** Representative photomicrographs of AsPC-1 and PANC-1 shNT and shECT2-2 cellular invasion through Matrigel-coated chambers; n=3. **D** Representative photomicrographs of single PDAC shNT and shECT2-2 spheres and clonal expansion efficiency of AsPC-1 and PANC-1 CSCs in nonadherent culture. Results presented as % of single CSCs that expanded or did not expand; n =25. **E.** Oncosphere size of AsPC-1 and PANC-1 shNT and shECT2-2 cells, normalized to their respective shNT control. Quantitated results in A-C and E expressed as % NT control and represent the mean ± SEM. (*) *p* < 0.05 compared to NT.

**Figure S3. ECT2 is required for PDAC cell tumorgenicity *in vivo* and associates with PDAC cell proliferation in tumors. A** Tumor growth curve of subcutaneous tumor bearing NOD-SCID mice inoculated with MiaPaCa-2 shNT or shECT2 cells, using two different ECT2 shRNA constructs (shECT2-1, and -2); n=6 mice/group. **B** Final weights of tumors in panel A at harvest (42 days) express as mean weight ± SEM. **C** Final bioluminescence of PANC-1 shNT or shECT2 orthotopic tumors. Results represent as the mean bioluminescence ± SEM. n=7 mice/group **D** Representative bioluminescent images of primary and metastatic PANC-1 shNT tumors. **E** and **F** ECT2 IHC in shNT and shECT2 MiaPaCa-2 subcutaneous and PANC-1 pancreas orthotopic tumors. **G** and **H** Ki67 IHC in shNT and shECT2 MiaPaCa-2 subcutaneous and PANC-1 pancreas orthotopic tumors. **I** and **J** CD31 IHC in shNT and shECT2 MiaPaCa-2 subcutaneous and PANC-1 pancreas orthotopic tumors. Quantitated results in E-J represent the mean ± SEM and expressed as % NT control in panels. (*), *p* < 0.05.

**Figure S4. ECT2 knockdown does not impair PDAC anchorage dependent cell growth.** **A** Growth curve of AsPC-1, CFPAC-1, and MiaPaCa-2 shNT and shECT2 cells over 7 days; n=5 **B** Representative DAPI (blue) and phalloidin (red) immunofluorescence to visualize multinucleated cells in shNT and shECT2 cells.

**Figure S5. Nuclear and cytoplasmic ECT2 support PDAC transformation A** Expanded field of view of Figure 5A. **B** representative immunofluorescence image of ECT2 (green) cellular localization in AsPC-1 and MiaPaCa-2 cells. **C** Immunoblot analysis of ECT2 in cytoplasmic (C) and nuclear (N) fractions in PANC-1 shECT2 knockdown cells expressing HA-tagged WT, or NLS^mut^ ECT2 or vector (Vec). Lamin A/C and MEK-1 served as control for the nuclear and cytoplasmic fractions, respectively. **D** Immunoblot analysis of AsPC-1 shECT2 knockdown cells expressing HA-tagged WT, or NLS^mut^ ECT2 or vector (Vec). B-actin served as loading control. Effects of WT or NLS^mut^ ECT2 reconstitution in AsPC-1 shECT2 knockdown cells on **E** transformed growth; n=5 and **F** invasion through Matrigel-coated chambers; n=3. Results expressed as % shNT and represent the mean ± SEM. **G** Effects of WT or NLS^mut^ ECT2 reconstitution in AsPC-1 shECT2 knockdown cells on CSC clonal efficiency. Results presented as % of single CSCs that expanded or did not expand; n = 25. **H** CSC sphere size expressed as mean diameter in micrometers ± SEM; n = 25. **I** qPCR analysis of 45S rRNA in AsPc-1 shECT2 knockdown cells expressing vector (Vec), or WT, or NLS^mut^ ECT2. Results expressed as % NT and represent the mean ± SEM. (*) p < 0.05 compared to shNT and (**) p < 0.05 compared to vector in panels **E-I**.

**Figure S6. ECT2 GEF activity is required for PDAC transformation.** **A** Immunoblot analysis for total ECT2 and HA-ECT2 in AsPC-1 shECT2 knockdown cells expressing with vector (Vec), WT, or DH^mut^ ECT2. B-actin served as loading control. Effects of WT or DH^mut^ ECT2 expression in AsPC-1 shECT2 knockdown cells on **B** transformed growth; n=5 and **C** invasion through Matrigel-coated chambers; n=3. Results expressed as % shNT and represent the mean ± SEM. **D** Effects of WT or DH^mut^ ECT2 expression in AsPC-1 shECT2 knockdown cells on CSC clonal efficiency. Results presented as % of single CSCs that expanded or did not expand; n = 25. **E** CSC sphere size expressed as mean diameter in micrometers ± SEM; n = 25. (*) *p* < 0.05 compared to NT.

**Figure S7. ECT2 mediates MEK/ERK signaling in PDAC cells.** **A** Phosphorylated and total ERK1/2 IHC in PANC-1 shNT and shECT2 pancreas orthotopic tumors. **B** Immunoblot analysis of phosphorylated and total MEK1/2 and ERK1/2 levels in vector (Vec) or WT, DH^mut^ or NLS^mut^ ECT2 expressing AsPC-1 shECT2 knockdown cells. B-actin served as loading control. **C** Immunoblot analysis of the effects of expression of constitutively active Rac1 (caRac1V12) in PANC-1 shECT2 knockdown cells on the levels phosphorylated and total MEK1/2 and ERK1/2. B-actin served as control. **D** Immunoblot analysis of the effects of expression of constitutively active RhoA (caRhoAV17) in PANC-1 shECT2 knockdown cells on the levels of phosphorylated and total LIMK, MYPT-1 and MLC2. B-actin served as control. **E** Immunoblot analysis of the effects of expression of constitutively active Rac1 (caRac1V12) in PANC-1 shECT2 knockdown cells on the levels of phosphorylated and total LIMK, MYPT-1 and MLC2. **F** Immunoblot analysis of the effects of expression of constitutively active RhoA (caRhoAV17) in PANC-1 shECT2 knockdown cells on the levels of phosphorylated and total MEK1/2 and ERK1/2 (*) *p*< 0.05 compared to NT.

**Figure S8. Correlation between ERK and ROCK pathway signatures and the expression of GEFs in human primary PDAC tumors. A** and **B** Scatterplots showing the correlation between VAV1, TIAM1, and DOCK8 mRNA expression and ERK and ROCK pathway signature scores, respectively, in the TCGA PDAC dataset (n= 179).

**Figure S9. Targeting of ECT2-RAC1 signaling enhances PDAC cell sensitivity to MEK inhibition.** **A.** Quantitation of relative colony formation in PDAC cells expressing shNT or shECT2 treated with increasing concentrations of Trametinib; n=5 * *p* <0.05 relative to vehicle treated control. **B.** Representative clonogenic assays of PDAC cell lines treated with increasing concentrations of ANF; n=5. Relative dose–response curves showing cell viability (**C**) and colony formation (**D**) following treatment with ANF, Trametinib, or the combination in PDAC cells; n=5; ** p* <0.05 relative to vehicle treated control and *** p* <0.05 relative to Trametinib alone treatment. **E**. Representative clonogenic assays of PDAC cell lines treated with increasing concentrations of NSC23766; n=5 **F.** Dose-response curves showing cell viability following treatment with NSC23766, Trametinib, or the combination in PDAC cells; n=5. **G.** Quantitation of relative colony formation in PDAC cells treated with NSC23766 and Trametinib alone or in combination. N=5 ** p* <0.05 relative to vehicle treated control and *** p <0.05* relative to Trametinib alone treatment**. H** Dose-response curves showing cell viability of PDAC cell lines treated with increasing concentrations of Y-27632.

**Table S1. Reagents and molecular tools used in this study.** **A** List of primary and secondary antibodies used for immunoblotting, immunohistochemistry, and immunofluorescence analyses, including supplier, catalog number, and RRID where available. **B** Target sequences of shRNA constructs used for ECT2 knockdown. **C** TaqMan assay IDs and primer sequences used for quantitative real time PCR (qPCR) analyses. Abbreviations: CDS, coding sequence; RRID, Research Resource Identifier.

**Table S2. Clinicopathologic characteristics of patients with PDAC.** Summary of the clinicopathologic features of the 26 PDAC patient samples included in this study, including age at diagnosis, sex, histologic differentiation, tumor stage, and recurrence status. Data are presented as median (range) or number of cases (%). UNK, unknown.

**Table S3. Genes associated with ERK, KRAS/ERK, and ROCK signaling pathway signatures.** List of genes comprising the ERK, KRAS/ERK, and ROCK signaling pathway gene signatures used for pathway enrichment analyses in PDAC tumors. The total number of genes included in each pathway signature is indicated. These gene sets were used to evaluate the association between ECT2 expression and activation of Rho GTPase and MAPK signaling pathways in human PDAC datasets.
